# Adaptive Gaze Strategies for Locomotion with Constricted Visual Field

**DOI:** 10.3389/fnhum.2017.00387

**Published:** 2017-07-27

**Authors:** Colas N. Authié, Alain Berthoz, José-Alain Sahel, Avinoam B. Safran

**Affiliations:** ^1^UPMC Université Paris 06, UMR S968, Institut de la Vision, Sorbonne Universités Paris, France; ^2^Institut National de la Santé et de la Recherche Médicale, U968, Institut de la Vision Paris, France; ^3^Centre National de la Recherche Scientifique, UMR 7210, Institut de la Vision Paris, France; ^4^Centre Hospitalier National d'Ophtalmologie des Quinze-Vingts, Institut National de la Santé et de la Recherche Médicale-DHOS CIC 1423 Paris, France; ^5^Equipe Pr Alain Berthoz Professeur Emérite au Collège de France Paris, France; ^6^Institute of Ophthalmology, University College London London, United Kingdom; ^7^Fondation Ophtalmologique Adolphe de Rothschild Paris, France; ^8^Department of Ophthalmology, School of Medicine, University of Pittsburgh Pittsburgh, PA, United States; ^9^Département des Neurosciences, Université de Genève Geneva, Switzerland

**Keywords:** retinitis pigmentosa, peripheral visual field loss, adaptation, orientation and mobility, gaze strategy, eye-head coordination

## Abstract

In retinitis pigmentosa (RP), loss of peripheral visual field accounts for most difficulties encountered in visuo-motor coordination during locomotion. The purpose of this study was to accurately assess the impact of peripheral visual field loss on gaze strategies during locomotion, and identify compensatory mechanisms. Nine RP subjects presenting a central visual field limited to 10–25° in diameter, and nine healthy subjects were asked to walk in one of three directions—straight ahead to a visual target, leftward and rightward through a door frame, with or without obstacle on the way. Whole body kinematics were recorded by motion capture, and gaze direction in space was reconstructed using an eye-tracker. Changes in gaze strategies were identified in RP subjects, including extensive exploration prior to walking, frequent fixations of the ground (even knowing no obstacle was present), of door edges, essentially of the proximal one, of obstacle edge/corner, and alternating door edges fixations when approaching the door. This was associated with more frequent, sometimes larger rapid-eye-movements, larger movements, and forward tilting of the head. Despite the visual handicap, the trajectory geometry was identical between groups, with a small decrease in walking speed in RPs. These findings identify the adaptive changes in sensory-motor coordination, in order to ensure visual awareness of the surrounding, detect changes in spatial configuration, collect information for self-motion, update the postural reference frame, and update egocentric distances to environmental objects. They are of crucial importance for the design of optimized rehabilitation procedures.

## Introduction

Peripheral visual loss impairs several mechanisms involved in the interaction with the environment, including those participating in motion discrimination (Turano and Wong, 1992) and spatial localization (Temme et al., 1985). From a visuo-motor perspective, the restriction of the peripheral visual field (VF) alters postural control (Turano et al., 1993; Berencsi et al., 2005) and leads to orientation errors during locomotion (Turano et al., 1998, 2002), resulting in unintentional contacts with objects (Peli et al., 2016). As a consequence, affected individuals limit independent travel (Turano et al., 1999) and walk slowly (Geruschat et al., 1998; Friedman et al., 2007). Human locomotion is a complex task involving multisensory integration, postural control, generation of locomotor trajectories relying on spatial orientation and motor control of the whole body in a coordinated way. Like subjects affected by central VF loss (Safran et al., 1999), patients who lose some of their peripheral VF should adapt their behavior and sensorimotor strategies. They may develop a variety of adaptive sensorimotor strategies, ranging from a revised gaze behavior (i.e., eye and head movements), to full body trajectory modifications.

In previous studies on behavioral changes in subjects with peripheral VF loss, we identified the following main forms of modifications. 1. An increased number of saccades and fixations, allowing a more active visual exploration of the environment (Coeckelbergh et al., 2002b; Crabb et al., 2010); this however was not corroborated by other studies showing no difference in fixation number with real (Wiecek et al., 2012) and simulated deficit (Cornelissen et al., 2005); or even a decrease in saccade rate (Smith et al., 2012a,b). 2. In subjects suffering from retinitis pigmentosa (RP), exploratory saccades were found larger in amplitude than in controls, leading to fixations beyond the VF limits (Luo et al., 2008). Moreover, among glaucoma patients, those performing saccades beyond their VF limits exhibited better search performances (Sippel et al., 2014). Conversely, other studies found that the saccade amplitude in affected patients was similar to that of controls during visual search (Smith et al., 2012a,b), or locomotion (Luo et al., 2008), while others even reported smaller amplitudes with simulated peripheral VF loss (Cornelissen et al., 2005). 3. Independently of saccade rate or amplitude, affected subjects could have a wider range of scanning of eye movements with respect to the head. Contrary to expectations, data indicated a reduction of the horizontal eye-position dispersion (Vargas-Martin and Peli, 2006). 4. Scanning the visual environment involved wider head movements in affected individuals during driving (Coeckelbergh et al., 2002a; Kasneci et al., 2014), but was not quantified during locomotion. 5. With peripheral VF loss, fixation area was found larger during locomotion (Turano et al., 2001), although they were smaller in another study on traffic gap judgment task (Cheong et al., 2008). 6. Finally, the sequence of fixations in the environment—where and when—was analyzed in an apparently single study (Turano et al., 2001), which showed that during locomotion, RP subjects frequently fixated downward, at objects, or at the layout.

Several factors could explain these conflicting results, including (i) heterogeneity in conditions underlying studied VF loss (i.e., glaucoma and RP^1^, or even simulated VF loss), (ii) variability in residual VF extent, (iii) possible confounding factor of associated visual acuity, (iv) limitation in sample size, and (v) variability in performed tasks. For instance, Luo et al. (2008) showed that the saccade rate was different for a single subject in a walking or a visual search task. Moreover, in most locomotor tasks, because of the ecological nature of the task, many variables were not systematically controlled: the goal direction, the lighting, the presence of various obstacles, the surrounding sounds, etc.

In order to reduce the number of running variables, the present study investigates the impact of peripheral VF loss on gaze strategies during a simple, goal-oriented locomotor task. Goal-directed locomotion has been extensively studied in healthy individuals, using motion-capture devices (Hicheur et al., 2007; Cinelli et al., 2009; Pham and Hicheur, 2009; Cinelli and Warren, 2012; Authié et al., 2015). Several attempts have been made to relate the stereotypy of the trajectories to geometrical laws or to optimization (Bennequin et al., 2009). The locomotor path was found to be similar across visual conditions (i.e., in either lighted or dark environment) and walking speed (Pham et al., 2011), leading to postulate a dissociation between sensorimotor and spatial cognition processes (Hicheur et al., 2016). A few studies where done on patients with vestibular loss (Takei et al., 1996; Glasauer et al., 2002). However, to our knowledge, the precise characteristics of trajectories performed by the visually impaired were never assessed.

To investigate that issue in a clinically homogeneous, well defined group of individuals, we carefully selected subjects suffering from retinitis pigmentosa, affecting primarily the peripheral VF, in a similar extend. RP is a rod-cone dystrophy leading to bilateral constriction of the VF and eventually, at most advanced stages, to complete blindness (Heckenlively et al., 1988). At intermediate stages, RP induces a “tunnel vision” that associates a peripheral VF loss with a relatively spared central area, and accordingly preserved visual acuity (VA). We selected patients exhibiting a residual VF from 10 to 25° in diameter, as <25° residual VF reportedly results in impaired mobility (Black et al., 1996; Haymes et al., 1996; Geruschat et al., 1998; Bowers et al., 2004; Hassan et al., 2007), and a sufficient VA (i.e., 20/40 or better) to investigate the behavioral changes specifically induced by peripheral VF loss.

The locomotion experiment was defined taking into account the following two constraints: to be ecological in nature, in order to directly illustrate difficulties encountered by RP patients in daily-life activities, and experimentally controlled to encompass spontaneous trajectory formation. The task therefore consisted of walking toward—and passing through an obliquely-oriented doorway, either with or without obstacle. We compared full body behavior of normally sighted subjects and RP subjects (RPs) with tunnel vision, using motion capture and eye movement measurements. Characteristics of the trajectory, gaze and body movements were assessed, and the sensorimotor compensatory mechanisms developed by both patients and controls were analyzed. To our knowledge this is the first study in which all these characteristics have been measured simultaneously with a high degree of precision.

## Materials and methods

### Subjects

**Figure 1:**
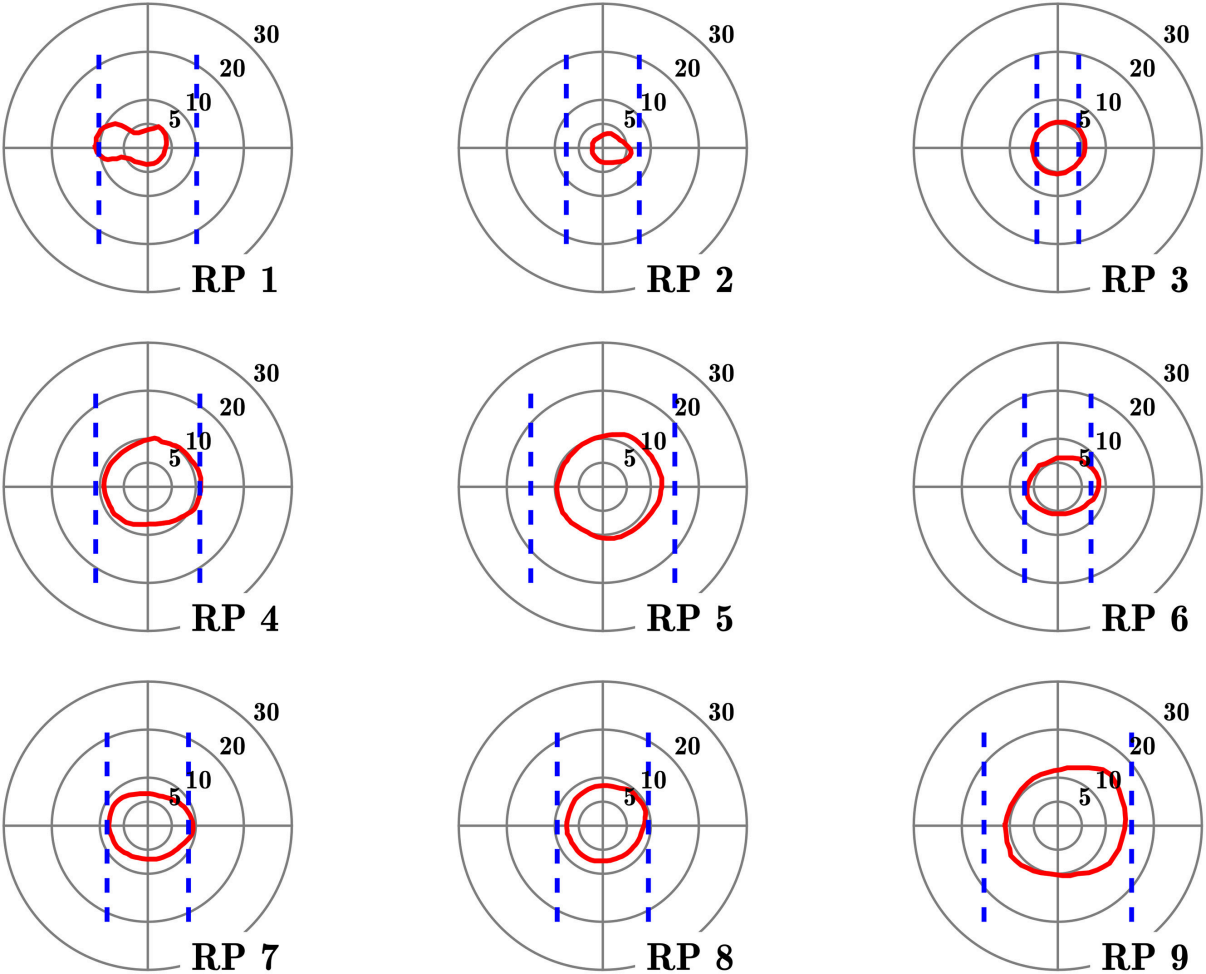
Visual fields of the subjects with retinitis pigmentosa. Red contours define isopter plotted with a Goldmann III/4 target, on a 5–30° concentric scale. Dashed blue lines define the seeing areas evaluated by door edge perception.

Two groups participated in the study:

- Nine subjects with RP (all right-handed, 6 males), presenting a central residual field from 10 to 25° in diameter (as measured by Goldmann III/4 kinetic perimetry; see Figure 1 and Table 1), with a best corrected visual acuity equal or superior to 20/40 (measured by ETDRS charts^2^). Ages ranged from 30 to 64 years (mean: 45 years).
- Nine control subjects with normal vision (all right-handed, 3 males), with a best corrected visual acuity equal or superior to 20/20 (ETDRS). Ages ranged from 29 to 60 years (mean: 45.9 years).

**Table 1:** Clinical characteristics of tested subjects.

**Subject**	**Age (yr)**	**Diagnosis**	**Visual Acuity**				**Visual field diameter Goldmann III/4e**	**Visual field diameter door borders**
			**Snellen**		**LogMAR**			
			**Left**	**Right**	**Left**	**Right**		
CO 1	33	Normal vision	20/16	20/16	−0.1	−0.1	−	−
CO 2	46	Normal vision	20/16	20/20	−0.1	0	−	−
CO 3	57	Normal vision	20/16	20/16	−0.1	−0.1	−	−
CO 4	40	Normal vision	20/16	20/16	−0.1	−0.1	−	−
CO 5	40	Normal vision	20/16	20/16	−0.1	−0.1	−	−
CO 6	44	Normal vision	20/16	20/16	−0.1	−0.1	−	−
CO 7	29	Normal vision	20/12.5	20/12.5	−0.2	−0.2	−	−
CO 8	56	Normal vision	20/20	20/20	0	0	−	−
CO 9	60	Normal vision	20/20	20/20	0	0	−	−
RP 1	57	RP	20/25	20/25	0.1	0.1	14.6° × 7.3°	20.37°
RP 2	53	RP	20/40	20/40	0.3	0.3	8.2° × 6.1°	15.2°
RP 3	30	RP	20/25	20/40	0.1	0.3	10.9° × 10.7°	8.72°
RP 4	32	RP	20/25	20/25	0.1	0.1	20.2° × 17.9°	21.7°
RP 5	53	RP	20/40	20/32	0.3	0.2	21.9° × 21.5°	30.01°
RP 6	62	RP	20/25	20/40	0.1	0.3	14.9° × 11.7°	13.84°
RP 7	32	RP	20/25	20/20	0.1	0	17.6° × 13.5°	16.97°
RP 8	30	RP	20/32	20/32	0.2	0.2	16.1° × 15.7°	18.99°
RP 9	64	RP	20/32	20/25	0.2	0.1	24.9° × 22.3°	30.75°

*Snellen and LogMAR visual acuity were measured in all individuals. RPs VF was first assessed by Goldmann III/4 kinetic perimetry (see also Figure 1); maximal horizontal × vertical diameter are reported here. Moreover, RPs horizontal VF diameter was evaluated based on the perception of door edges used in the task*.

Table 1 lists the clinical characteristics of tested subjects. No subject had any reported neurological or psychiatric disease. They were not taking neurotropic drugs. Groups were matched for age (no significant difference between groups observed, Mann–Whitney *U* = 39.5, *n*_1_ = *n*_2_ = 9, *p* = 0.96). Complementing Goldmann kinetic perimetry, in RP subjects, we determined the horizontal diameter of the VF, taking into account both ambient luminosity and actual appearance of spatial cues in the experimental setting. For that purpose, the horizontal VF was estimated by asking RP subject to fixate a target located in front of him and indicate when he first perceived the edge of the door frame used in the experiment (see below), that was progressively displaced from the side toward the central target, successively from the right and the left. VF horizontal angular dimension was then computed using subject and frame positions^3^.

The study was approved by the ethical committee (ANSM: 2014-A00812-45; CPP Île-de-France 14959) in accordance with the Declaration of Helsinki. Written informed consent was obtained from the subjects after explanation of the nature and possible consequences of the study.

### Recording apparatus

Motion capture of whole body kinematics has been conducted using a VICON system (VICON Motion Systems Inc., Los Angeles, 8 cameras, 120 Hz). Subjects wore a tight black suit allowing sticking the VICON markers close to the subject's body. Altogether, 43 markers were placed according to the “Plug-in Gait” model of the VICON system (VICON, V-1.7). To approximate head position, four additional markers were located over the right and left temples on the back and at the front of the head. Four markers were also positioned on each obstacle and door frame. Marker position were recorded, reconstructed and labeled using VICON Nexus 2.0 software.

**Figure 2:**
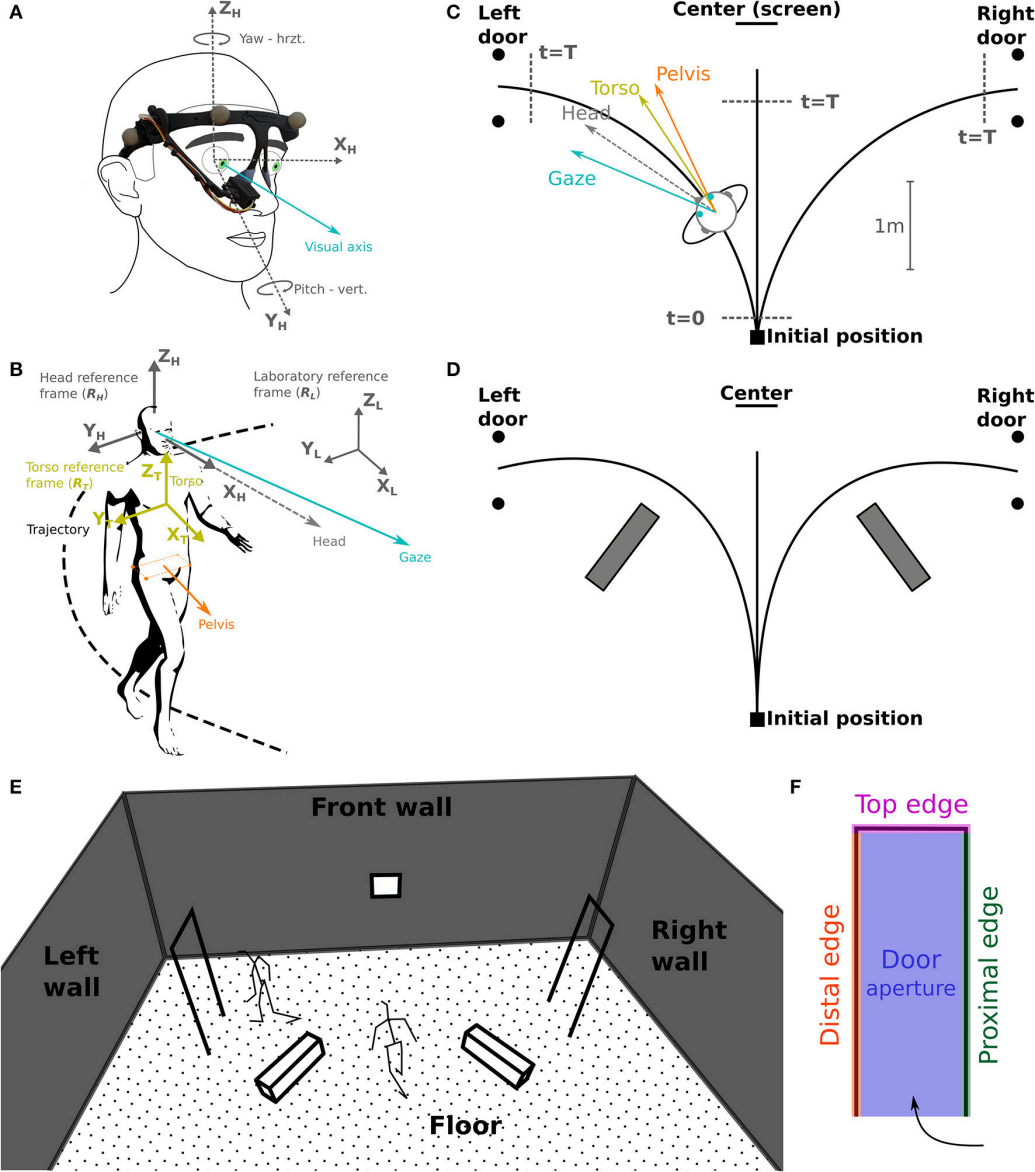
Task schematic representation and coordinates systems. **(A)** The camera of the eye-tracker, placed on the right eye, records eye movements (visual axis, blue) in azimuth and yaw. **(B)** Selected reference frames: laboratory (R_L_), head (R_H_) and torso (R_T_); considered segments: gaze, head, torso, and pelvis. **(C)** Schematic top view of the experimental set-up. Subjects were asked to reach either a central target (screen on the wall), the left or the right door. The walking start and stop time, *t* = 0 and *t* = T, respectively, are used for time normalization. **(D)** Indicative positions of the obstacles lying on the floor. **(E,F)** Gaze fixation categories: definition of 3D surfaces/volumes.

Eye movement recording was performed with a head-mounted video eye-tracker (Mocaplab, Paris, 60 Hz, mass: 30 g). The camera was located under subject's right eye, paying attention to avoid masking an important part of subject's field of vision (Figure 2A). The camera output was transmitted to a Microsoft Surface tablet (Microsoft Inc., Redmond, mass: 800 g), that was tied on subject's back. Temporal synchronization of eye-tracker and VICON signals was eventually performed off-line. At the beginning of the eye video recording, a sound signal was transmitted to an electrical circuit, triggering three infrared LEDs (780 nm wavelength, Thorlabs Inc., Newton) identified as markers by the VICON system, to synchronize the recordings. The system calibration, described in Authié et al. (2015), was performed at the beginning and at the end of the experiment.

### Task

Testing sessions took place in the Streetlab artificial street, an experimental room of 5.5 × 9.0 × 6.0 m (L × W × H) in size, with a controlled illumination of 235 lux. The subject started from a defined initial position and had to walk at a normal pace, either (a) straight ahead toward a visual target placed on the wall in front, or (b) through one of the two white doors, materialized by simple rectangular frames (0.73[W] × 2.05[H] m) located on the left or on the right side of the subject's initial position (Figure 2A). Two conditions were explored in separate blocks: (a) without obstacles, and (b) with obstacles consisting of white blocks (1.1[L] × 0.3[W] × 0.3[H] m) placed on the ground to force subjects to deviate from a direct trajectory to the door frame, in order to avoid touching them (Figures 2B,D). At the beginning of the trial, subjects were instructed to fixate a cross displayed at the center of a screen facing them. Then, a recorded voice indicated the direction to follow—i.e., left, center (straight-forward), or right. Coincidentally, an infrared LED was lit to mark the beginning of the trial. When requested to walk in the central direction, the subject had to stop just in front of the facing wall, wait for 1 s, and come back to the starting position. If asked to walk to—and pass through a lateral door, he had to keep walking beyond the door to come back to the starting position. Trials were randomized, with 14 trials including obstacles (6 leftward, 6 rightward, 2 straight-forward), and 38 trials without obstacles (15 leftward, 15 rightward, 8 straight-forward). To avoid diverting the subjects' attention, all walls were clothed with black curtains, and experimenters monitored the experiment from a separate room.

### Data analysis

**Figure 3:**
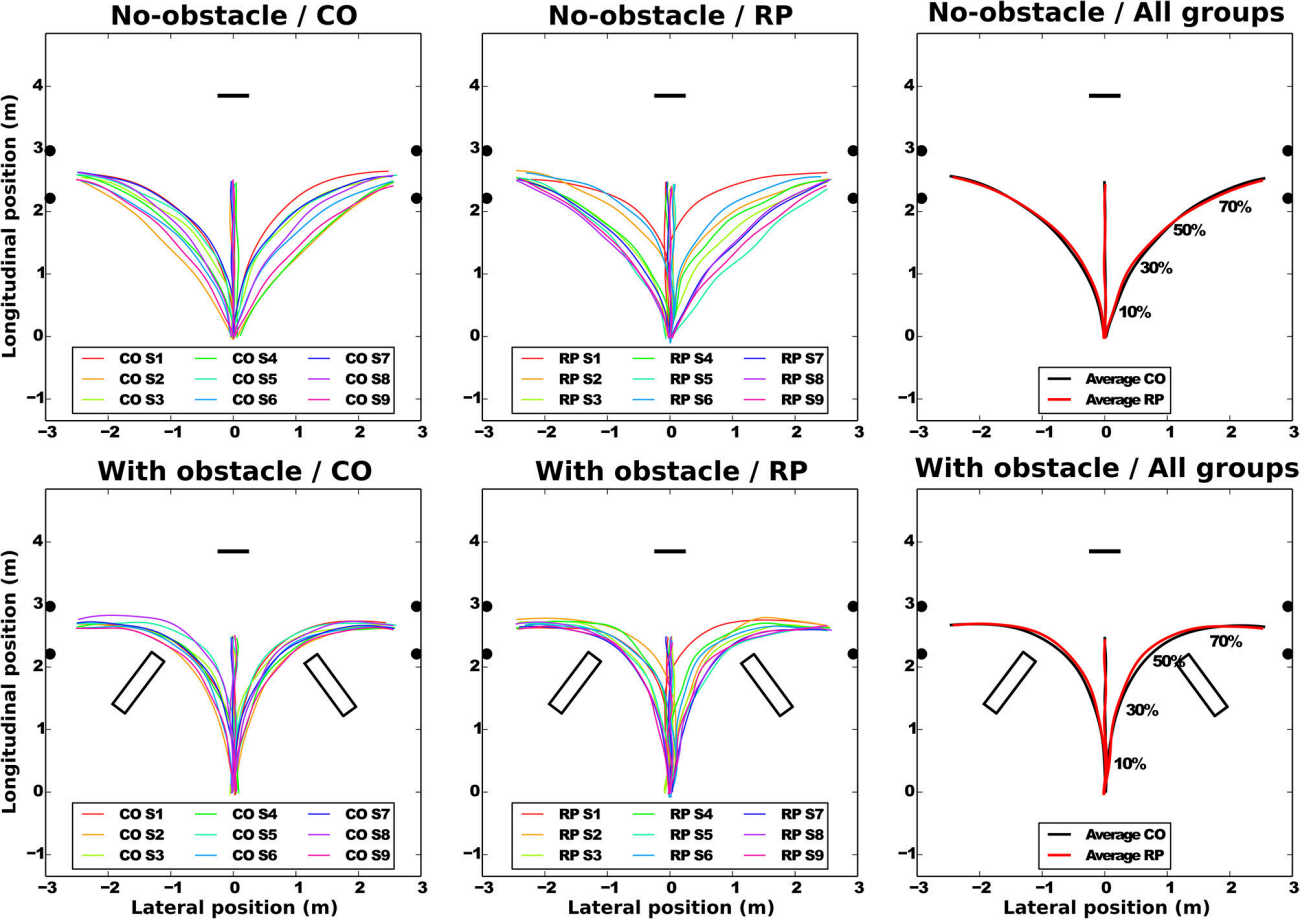
Average pelvis trajectories for all subjects in each experimental condition (**top:** *no-obstacle* condition; **bottom:** *with obstacles*). CO stands for control subjects. The horizontal black segment indicates the position of the screen; the black disks, the door edges; and the black rectangles, the obstacles. On the rightmost part of the figure, the normalized percentage of progression in the trajectory is shown.

Data analysis has been performed with Python 2.7.6 (http://www.python.org) equipped with NumPy 1.8.2 (http://www.numpy.org). Statistical analysis has been carried out with R 2.2.2 (http://www.R-project.org). Body motion capture data were Butterworth filtered with a cutoff frequency of 15 Hz (Bernardin et al., 2012, with a zero-phase procedure that did not induce lag in the signal). For each trial, three phases were considered: (1) the *waiting phase*, from the first fixation on the screen to the audio signal indicating the direction to follow, (2) the *preparation phase*, i.e., the subject started walking (12 cm from the starting point, *t* = 0, Figure 2C), and (3) the *walking phase*, from walk initiation to reaching 40 cm before the aimed door position or 85 cm before the screen (center, *t* = T, Figure 2C). Most variables were computed only during the walking phase of the trial, unless notification in the text. All data were expressed in a right-handed space-fixed laboratory reference frame (Figures 2A,B). The head (R_H_) and torso (R_T_) reference frames were defined as in a previous study (Authié et al., 2015) from the rotations of rigid bodies given in terms of the Euler angles around the body-fixed axes in a Fick sequence (**Figure 6A**).

Before conducting eye movement analysis, phases corresponding to blinks were automatically excluded. We included in a single group all rapid-eye-movements (REM), i.e., both saccades or fast phases of nystagmus, as they cannot be automatically distinguished with the frame-rate of the eye tracker (Ron et al., 1972; Garbutt et al., 2001). We considered as REMs ocular movements that were at least 1° in amplitude, 30°/s in speed and 700°/s^2^ in acceleration peak (van der Steen and Bruno, 1995; Authié and Mestre, 2011). REM onset and offset were determined using an algorithm maximizing REM's amplitude (Authié et al., 2015), and were then manually checked.

### Relevant parameters

Subject's trajectory was assessed based on the position of pelvis isobarycenter (Bernardin et al., 2012; Authié et al., 2015), and computed for determining trajectory length and walking speed. To investigate eye movements in the head reference frame, we computed for each trial the REM number, rate (number of REMs per second) and amplitude. For RP subjects, we also quantified the proportion of REMs larger in amplitude than VF diameter (so-called REM beyond the VF; Luo et al., 2008). Moreover, as in Vargas-Martin and Peli (2006) study, we computed the standard deviation of horizontal and vertical eye movement in R_H_, providing the dispersion range of eye movements. Head orientation (yaw, pitch, roll) was computed in R_T_: mean and standard deviation, essentially to assess the head stability. Finally, describing the fixation strategy in the environment required to pinpoint fixation phases, i.e., periods between two REMs, that were 83 ms or more in duration, corresponding to 5 successive frames in a 60 Hz recording (Turano et al., 2001). For each fixation, we computed the 3-D intersection between the gaze vector and the surfaces and volumes of the environment, i.e., the floor, the front wall including the screen, the obstacles, the doors frame, that was divided into four categories: proximal edge, distal edge, top edge, and aperture of the door (Figures 2E,F). To take into account measurement errors, we considered the intersections between the objects and a one-degree radius cone pointing from the eye center. For most considered variables, the data set used is available in a Supplementary File.

### Statistics

Repeated analyzes of variance (ANOVA, type II error) have been performed in order to assess the effect of the group (RP, control), obstacle (with or without), and trajectory direction (leftward, rightward, straight ahead). A threshold of *p* < 0.05 has been considered significant. Tukey's HSD tests have been used for *post-hoc* analysis whenever necessary. For non-normally distributed variables, Wilcoxon and Mann–Whitney tests with Bonferroni corrections for multiple comparisons were applied.

## Results

### Global trajectory performance

#### Trajectory kinematics

All subjects succeeded to perform the task without touching neither the obstacle nor the door frame edges. Pelvis trajectories are represented in Figure 3 for all groups.

**At the group level** (Figure 3, left and center), the trajectories demonstrate a sizable variability from group average trajectory for both controls (SD = 5.99 cm) and RPs (SD = 7.78 cm). The mean distance between the subject-averaged and the group-averaged trajectories revealed that the between-subject variability was lower with (7.89 ± 0.25 cm) than without obstacle (14.78 ± 0.73 cm, Table 2).

**Table 2:** Pelvis trajectory, eye variability, and head movements.

**Variable (unit)**	**Group**	**No-obstacle**			**With obstacles**			**Group effect**			**Obstacle effect**			**Direction effect**		
		**Leftward**	**Center**	**Rightward**	**Leftward**	**Center**	**Rightward**	**F**	***p***	**η^2^**	**F**	***p***	**η^2^**	**F**	***p***	**η^2^**
Trajectory variability (within group, cm)	Control	11.59 ± 5.96	–	15.05 ± 7.63	7.63 ± 3.37	–	6.45 ± 2.51	1.2	0.28	–	**21**	<**0.001**	**0.57**	1.8	0.19	–
	RP	13.08 ± 6.2	–	17.28 ± 9.44	9.05 ± 3.42	–	10.57 ± 8.14									
Trajectory variability (within subject, cm)	Control	4.58 ± 2.55	–	5.33 ± 1.89	2.3 ± 0.63	–	2.39 ± 0.97	0.2	0.69	–	**48**	<**0.001**	**0.75**	1.8	0.19	–
	RP	4.02 ± 0.74	–	4.29 ± 1.27	2.67 ± 1.09	–	2.89 ± 0.82									
Trajectory length (m)	Control	3.81 ± 0.15	2.43 ± 0.02	3.82 ± 0.25	4.26 ± 0.15	2.43 ± 0.01	4.29 ± 0.13	1.6	0.29	–	**197**	<**0.0001**	**0.92**	**986**	<**0.001**	**0.98**
	RP	3.91 ± 0.23	2.45 ± 0.04	3.88 ± 0.32	4.36 ± 0.14	2.44 ± 0.07	4.39 ± 0.22									
Walking speed (m/s)	Control	1.12 ± 0.08	0.97 ± 0.11	1.13 ± 0.09	1.13 ± 0.06	0.97 ± 0.12	1.14 ± 0.07	**14**	<**0.005**	**0.47**	**76**	<**0.0001**	**0.82**	0	0.83	–
	RP	0.95 ± 0.13	0.79 ± 0.11	0.96 ± 0.13	0.96 ± 0.12	0.78 ± 0.16	0.95 ± 0.11									
REM rate (Hz)	Control	2.52 ± 0.54	1.25 ± 0.57	2.56 ± 0.4	2.68 ± 0.48	1.18 ± 0.72	2.51 ± 0.4	**9.7**	<**0.05**	**0.38**	2.5	0.12	–	**84**	<**0.001**	**0.84**
	RP	3.04 ± 0.6	2.33 ± 0.84	3.07 ± 0.5	3.15 ± 0.54	2.65 ± 0.83	3.12 ± 0.52									
Vertical eye Variability (Hz)	Control	6.47 ± 2	6 ± 4.52	6.66 ± 2.36	6.78 ± 1.62	5.2 ± 4.81	7.71 ± 2.02	1.9	0.19	–	0.6	0.43	–	0.2	0.84	–
	RP	7.45 ± 2.39	8.53 ± 3.88	7.8 ± 2.56	7.27 ± 3	9.9 ± 3.78	7.16 ± 2.64									
Horizontal eye Variability (Hz)	Control	6.82 ± 2.4	1.94 ± 0.91	7.49 ± 2.29	6.73 ± 2.71	2.03 ± 1.02	7.21 ± 3.08	4.1	0.59	–	0.4	0.54	–	**71**	<**0.001**	**0.81**
	RP	8.79 ± 2.36	2.19 ± 0.9	9 ± 2.11	9.02 ± 2.64	3.75 ± 2.89	8.86 ± 2.54									
Mean head Yaw (°)	Control	−11.52 ± 4	0.04 ± 3.38	12.6 ± 6.9	−14.13 ± 6.1	−0.53 ± 2.81	13.34 ± 6.07	< 0.1	0.98	–	0.8	0.38	–	**164**	<**0.001**	**0.91**
	RP	−13.08 ± 11.1	−0.11 ± 9.92	13.13 ± 11.9	−14.6 ± 12.2	0.14 ± 8.22	14.76 ± 10.9									
Mean head Pitch (°)	Control	1.5 ± 7.3	−3.96 ± 4.96	1.4 ± 7.02	3.5 ± 8.41	−3.48 ± 6.76	2.09 ± 8.35	**15**	<**0.005**	**0.49**	**14**	<**0.005**	**0.45**	**68**	<**0.001**	**0.81**
	RP	14.5 ± 7.19	3.67 ± 5.62	14.53 ± 7.12	17.95 ± 7.91	5.03 ± 4.81	17.08 ± 7.51									
Head yaw Variability (°)	Control	4.36 ± 1	1.98 ± 0.47	4.08 ± 0.98	4.55 ± 0.9	1.92 ± 0.39	4.39 ± 1.05	**6.8**	<**0.05**	**0.3**	**8.8**	<**0.05**	**0.35**	**49**	<**0.001**	**0.75**
	RP	6.18 ± 2.75	1.95 ± 0.54	6.76 ± 3.17	8.06 ± 3.87	2.6 ± 1.31	7.88 ± 3.48									
Head pitch Variability (°)	Control	3.69 ± 1.6	2.76 ± 1.53	3.66 ± 1.42	3.72 ± 1.15	3.9 ± 2.38	3.73 ± 1.46	3.9	0.06	–	2	0.17	–	1.5	0.24	–
	RP	5 ± 1.73	5.3 ± 3.01	5 ± 1.94	4.07 ± 1.13	7.39 ± 5.01	4.08 ± 0.86									

*Mean values and between-subject standard deviation in control and RP participants for all directions and obstacle conditions. Anovas F-values, significance level (p) and effect size (η^2^) for each main effect. The degrees of freedom are (1, 16) for group and obstacle effects; and (2, 32) for direction, with an exception for the first two variables having identical degrees of freedom (1, 16) for each effect. Significant interactions are reported in the text*.

**At the subject level**, the between-trial/within-subject variability (standard deviation between trials and the subject-averaged trajectories, Table 2) was not statistically different between groups or directions (center direction excluded) and was lower with obstacles (2.46 ± 0.16 cm) than without obstacle (4.66 ± 0.35 cm).

Group-averaged trajectories are modified by the obstacle; the trajectory was more curved and longer with than without obstacle (Table 2, Figure 3). However, the trajectory length was identical between groups, even around the obstacles, as minimal distance between the obstacle corner and the pelvis trajectory was the same in both groups (RP: 0.42 ± 0.11 m; CO: 0.37 ± 0.09 m, Wilcoxon *Z* = 60, *n*1 = *n*2 = 9, *p* > 0.2).

**To summarize**, while in each group, for no-obstacle condition, we observed some differences of averaged trajectories between subjects, these differences were reduced in presence of an obstacle. This is captured by the between-subject variability, less similar than within-subject variability, which is equivalent between groups, and lower with obstacles. Moreover, the group-averaged trajectory was identical between groups, even around the obstacles.

#### Walking speed

As reported in Table 2, the walking speed was not influenced by the presence of obstacle, but controls walked faster than RPs. Both groups walked faster in the direction leftward (1.04 ± 0.1 m/s) and rightward directions (1.04 ± 0.1 m/s) than straight-ahead (0.87 ± 0.1 m/s). This could be related to a shorter length of trajectory toward the center (Table 2).

### Eye movements

**Figure 4:**
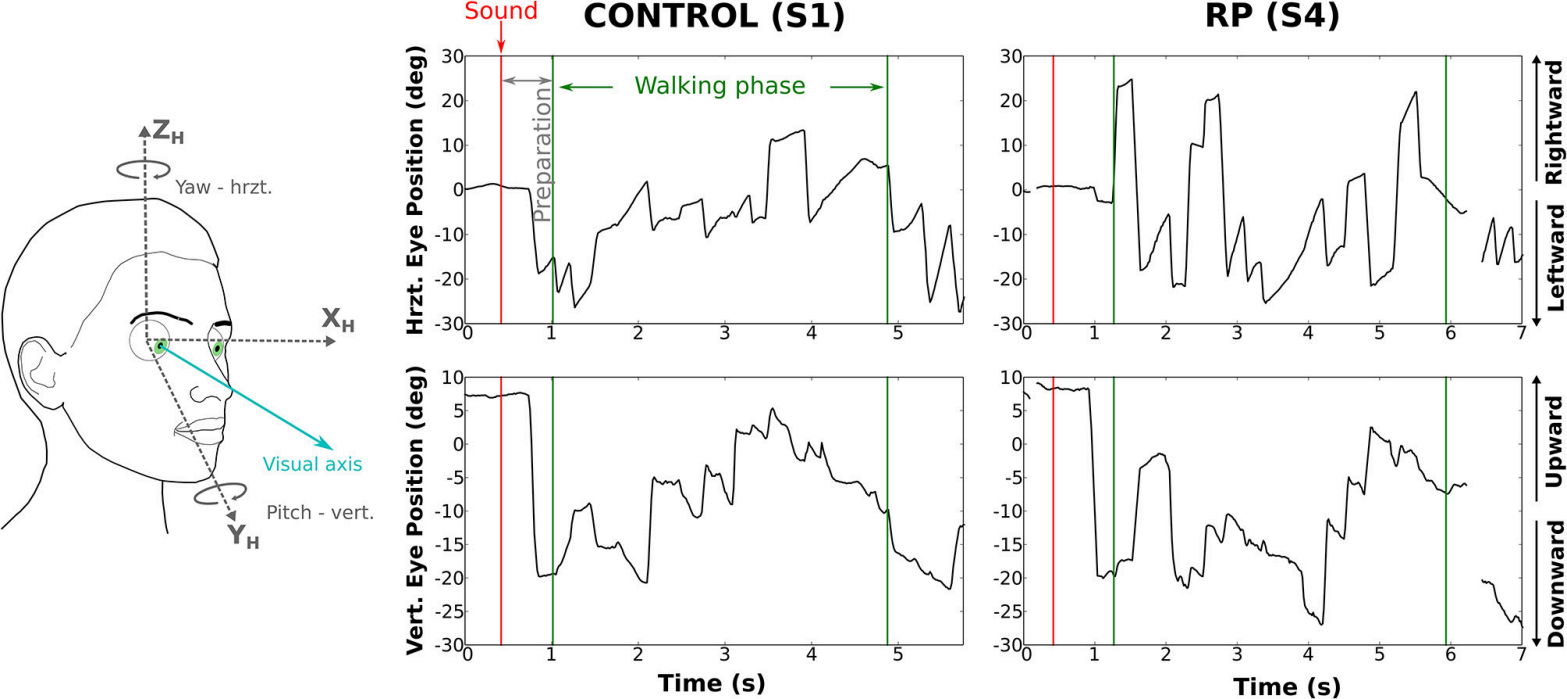
Rotations of the eye in the orbit of one control subject **(left)** and one RP subject **(right)** walking to the left door, with obstacle. The upper part of the figure corresponds to horizontal component and the lower part to the vertical. The red line marks the time the auditory instruction was given (i.e., end of the waiting phase), whereas green lines indicate the time limits of the walking phase.

We analyzed the eye movements during the walking phase, focusing on number, rate, and amplitude of rapid eye movements (REMs, Figure 4).

#### Number of REMs

Over all trials and conditions, 9,320 REMs were detected. Subjects made on average 9.4 ± 4.1 REMs per trial (CO: 7.2 ± 1.3; RP: 11.7 ± 2). As the trial duration was not equivalent for each group and experimental condition, we used REM rate (i.e., number of REMs per second) as an indicator of the exploratory behavior.

#### REM rate

**Figure 5:**
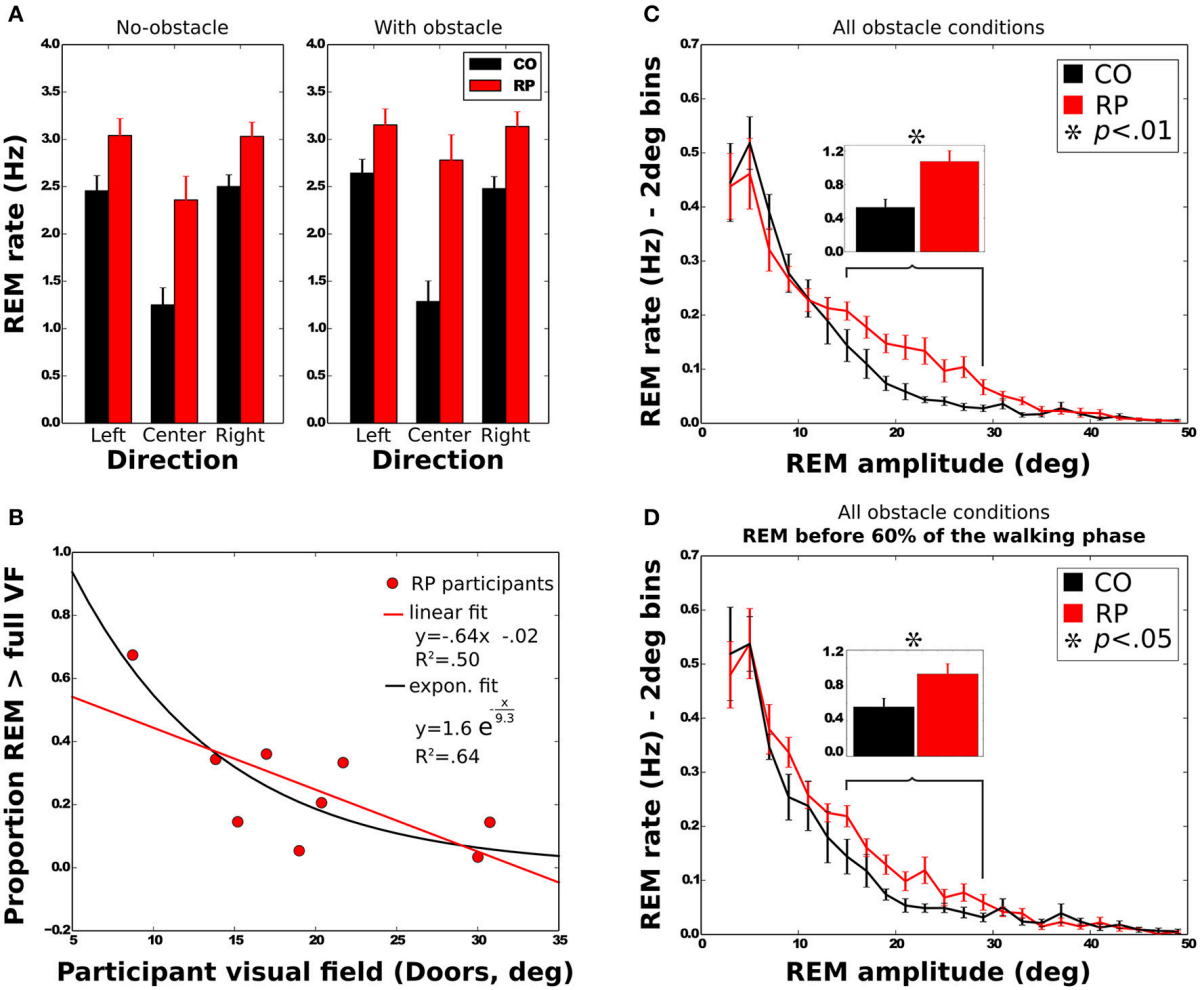
Characteristics of *rapid eye movements (REMs)*. **(A)** REM rate of control and RP subjects in each experimental condition, with between-subject standard error. **(B)** Mean proportion of REMs beyond the VF for RP subjects as a function of the VF with linear and exponential fits. **(C,D)** Distribution of the number of REMs per second as a function of the REM amplitude. Error bars are between-subjects standard error. In **(C)**, REMs for the whole trial were considered, although in **(D)** only REMs before 60% of the walking phase were taken into account. Histograms on top show the sum of the REM rate between 15 and 29° for all experimental conditions.

REM rate has been computed for each trial of each subject, and then averaged over trials (Table 2, Figure 5A). Anova analysis revealed that REM rate is higher in RPs (2.89 ± 0.58 Hz) than in controls (2.11 ± 0.45 Hz), and larger in the side than in the center direction for both groups (Center: 1.85 ± 0.63 Hz, Leftward: 2.85 ± 0.56 Hz, Rightward: 2.81 ± 0.52 Hz). The REM rate was larger in left and right than in center direction for both groups [group × direction interaction; *F*_(2, 32)_ = 12.27, *p* < 0.001, η^2^ = 0.44]. The presence of obstacles on the path did not affect REM rate.

#### REM amplitude

Note worthily, as shown in Figure 5C, in RPs REM are significantly more frequent than in controls in—and only in—the amplitude range from 15 to 29° (CO: 0.53 ± 0.30 Hz; RP: 1.07 ± 0.39 Hz; Mann–Whitney *U* = 12, *n*_1_ = *n*2 = 9, *p* < 0.01 two-tailed).

REM amplitude vary during the trial (**Figure 9B**), with RPs REM amplitude increased starting from the 60% of the trajectory, and peaking between 75 and 85% of the trajectory (~18°). It was significantly larger in RPs than in controls between 75 and 85% of the trajectory (Mann–Whitney *U* = 0, *n*_1_ = *n*2 = 9, *p* < 0.01 two-tailed). This occurred both in obstacle and no-obstacle conditions.

This indicated a large difference in oculomotor strategy when approaching the doors. Indeed, by the end of the trajectory, while controls tended to fixate the proximal edge of the door or in the door aperture (see also **Figure 10**), RPs alternately fixated proximal and distal edges (see also **Figure 11**), presumably as both edges could not be simultaneously perceived within the residual VF. To corroborate that hypothesis, we identified the sequences of at least three consecutive fixations alternating between proximal and distal door edges. This behavior was indeed much more frequent in RPs than in controls (RP: 54% of the trials; CO: 16% of the trials; *U* = 1, *n*_1_ = *n*2 = 9, *p* < 0.001 two-tailed Mann–Whitney).

We then considered the possibility that higher REM rate in RPs in the amplitude range from 15 to 29° (see above) could be related to this fixation strategy. However, we found that this difference remained significant when considering only the first 60% of the trajectory, corresponding to the trajectory before the end of obstacle circumvention (CO: 0.56 ± 0.30 Hz; RP: 0.93 ± 0.35 Hz; Mann–Whitney *U* = 17, *n*_1_ = *n*2 = 8, *p* < 0.05 two-tailed, see Figure 5D).

**In summary**, REM with an amplitude between 15 and 29° were more frequent in RPs than controls, even at the beginning of the trajectory. Moreover, as a result of a different gaze fixation strategy in RPs—i.e., *alternated fixations between door edges*—REM amplitude increased at the end of the trajectory.

#### REM beyond the VF

To further investigate the larger REM amplitude observed in RPs than controls, we computed the proportion of REMs that were larger in amplitude than half of the VF width (i.e., where post-saccadic fixation location had not been included within the VF at the pre-saccadic stage) and REM that were larger than the VF width (i.e., when pre- and post-saccadic VF did not overlap at all). All measured VFs were considered in the regression analyses (horizontal and vertical Goldmann VF, and door VF), but the reported values in this section were computed using the door VF, unless otherwise specified. On average, 49 ± 20% of the REMs were beyond half of the VF, and 25 ± 18% were beyond the full VF. A two-way Anova shows that the presence of obstacle did not influence the proportion of REMs beyond the VF [half: *F*_(1, 8)_ = 3.29; full: *F*_(1, 8)_ = 3.27; all *n.s*.]. The proportion of REMs beyond full VF was also identical for all trajectory directions [*F*_(2, 16)_ = 0.53, all *n.s*.], while REM proportion beyond half VF was larger in leftward (58%) and rightward (55%) than in straight ahead directions [34%; *F*_(2, 16)_ = 13.76, *p* < 0.001]. We also examined the correlation between the VF width and the proportion of REMs beyond the full VF (Figure 5B). Results showed that proportion of REMs beyond the full VF was significantly anti-correlated with the VF, with either door (*R* = −0.71) or horizontal Goldmann measures (*R* = −0.68; all *p* < 0.05). Results however were not significant when considering the vertical Goldmann measure (*R* = −0.55, *n.s*.). The same analysis with the half of VF leads to insignificant results.

**In summary**, larger REMs in RPs than controls often resulted in post-saccadic fixation beyond the limit of the VF, regardless of the obstacle condition. Moreover, for RPs, REM beyond the VF were more often performed by participants with a small VF.

#### Eye position variability

To provide accurate information about eye eccentricity from primary position, we computed the standard deviation of horizontal and vertical eye position in head reference frame. This eye position variability quantifies the dispersion of eye movements during walking (Vargas-Martin and Peli, 2006). Anova analysis showed no effect of all experimental factors on vertical variability of eye position (see Table 2). Horizontal variability of eye position didn't either differ between groups, even if one can note a tendency (Table 2). As expected, the horizontal variability was larger in the side than in the center direction. Finally, the horizontal variability was not influenced by the obstacle condition.

### Head orientation and variability

**Figure 6:**
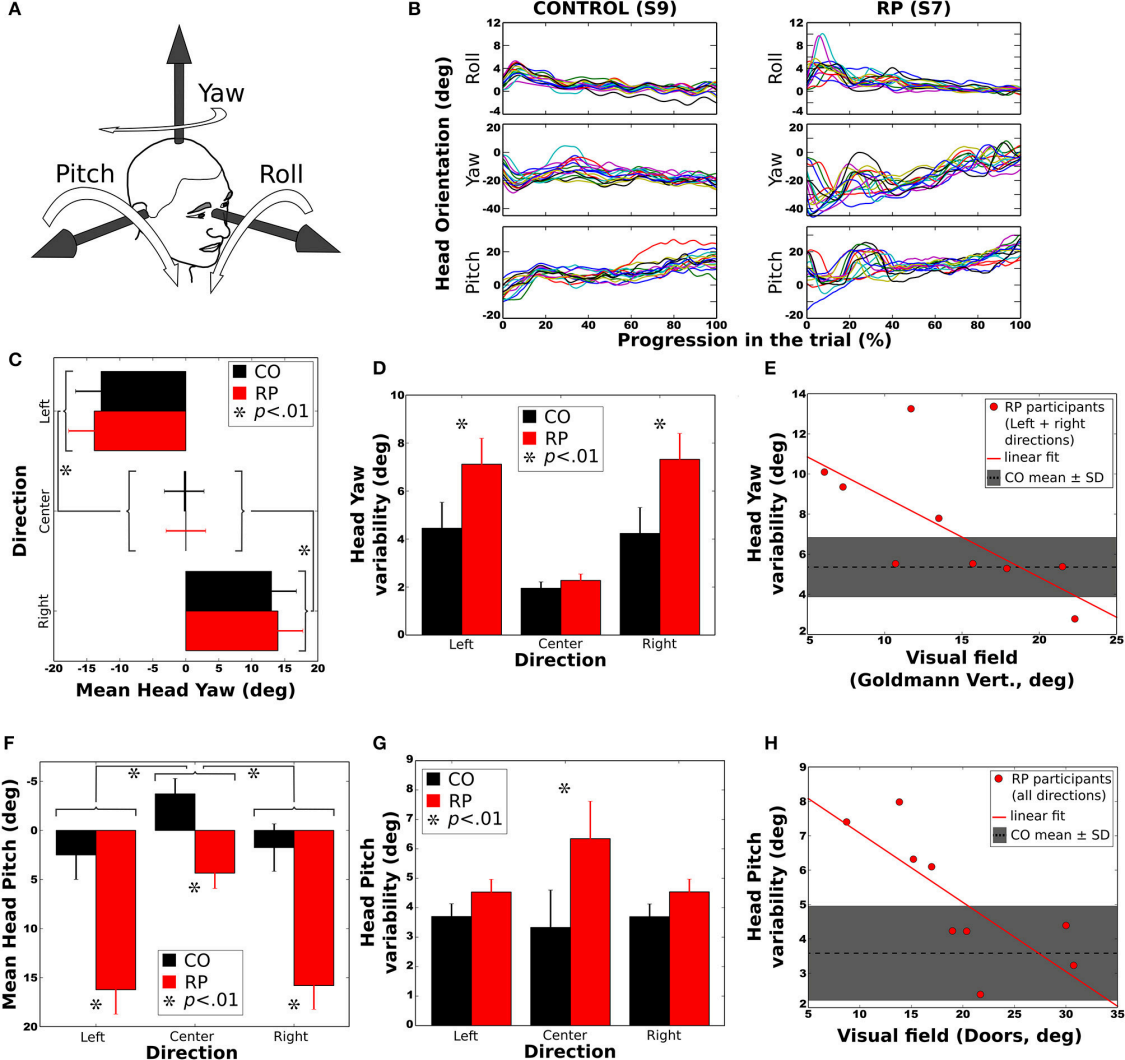
Head orientation during the trial. Error bars on figures **(C,D,F,G)** correspond to the between-subject standard error of the mean. Stars (^*^) indicates significant differences between groups or conditions. **(A)** Sign conventions for head axes of rotation. **(B)** Evolution of head orientation in all trials to the right and without obstacle, for a control subject (S9, left) and a RP subject (S7, right). **(C)** Mean head yaw did not differ between groups. **(D)** Head yaw variability (i.e., standard deviation during a trial) was larger for RP than for controls in left and right trajectory directions. **(E)** Head yaw variability was significantly anti-correlated to the residual VF of RPs (for leftward and rightward trajectory directions), larger VF leading to lower yaw variability. **(F)** Mean head pitch in all trajectory directions for both groups. Forward head tilt was more pronounced in RPs than in controls. **(G)** Head pitch variability (i.e., standard deviation during a trial) in all directions for both groups. Pitch variability was larger for RPs in the center direction. **(H)** Head pitch variability according to VF, for all trajectory conditions. As for yaw, larger VF led to smaller pitch variability. In both **(E,H)** the mean and standard deviation of head variability is indicated by a dashed line and a shaded area.

To determine whether RP subjects developed changes in head orientation, we investigated relationships between the trajectory direction and the head roll, yaw and pitch angles (with respect to the torso, see Figure 6A). Head roll did not vary much and was not considered for statistical analysis (Figure 6B). Head yaw was consistently in the direction of the trajectory (Figure 6C). The head pitch was most of the time oriented downward (Figure 6F), except for controls in straight ahead direction. The standard deviation of yaw and pitch angles was taken as a measure of the head variability during the trajectory.

#### Mean yaw

Analysis of variance indicated an effect of the trajectory direction but no effect of group or obstacle condition (see Table 2). Post-hoc showed that head yaw was oriented to the right in right curves and vice versa (Figure 6C). However, when head orientation was taken in absolute value, the difference between left and right trajectories was no longer significant (*p* > 0.20).

#### Yaw variability

Analysis of variance and *post-hoc* revealed that the head variability was larger for RPs than controls (see Figure 6D), larger for lateral trajectories than in straight one, and larger with than without obstacles. Moreover, the head yaw variability of RPs heading to the sides was significantly anti-correlated with their residual VF (Goldmann vertical: *R* = −0.72; all *p* < 0.05), indicating that head orientation varied more for subjects with a small residual VF (Figure 6E).

#### Mean pitch

Statistical analysis showed that the head was more oriented downward in RPs than in controls (see Figure 6F), more for lateral than straight-ahead trajectories, and more with obstacles than without. The head pitch and the residual VF in RPs were not significantly correlated (all *p* > 0.1).

#### Pitch variability

An interaction between groups and trajectory direction [*F*_(2, 32)_ = 3.41, *p* < 0.05, η ^2^ = 0.17] showed that head variability was larger for RPs in straight-ahead tasks that in all other conditions. Finally, the head pitch variability of RPs was significantly anti-correlated with their residual VF (Doors: *R* = −0.76; all *p* < 0.05), indicating that head orientation was less stable for subjects with a small residual VF (Figure 6H). In contrast, the head variability was not statistically different between groups (Figure 6G), trajectory directions and obstacle conditions.

**To summarize**, head yaw was, in average, more variable in RP than in control group, and for both groups more variable in leftward/rightward than straight-ahead directions, and with than without obstacles. Strikingly, the head was more oriented downward in RPs than in controls. Moreover, for RPs, head position in pitch varied more, and the head pitch was less stable for subjects with a small residual VF.

### Gaze orientation strategies

#### Horizontal and vertical angles between gaze and pelvis directions

In addition to the variability of eye and head position, we computed both horizontal and vertical distributions of the angle between the gaze and pelvis directions.

##### Horizontal gaze angle

**Figure 7:**
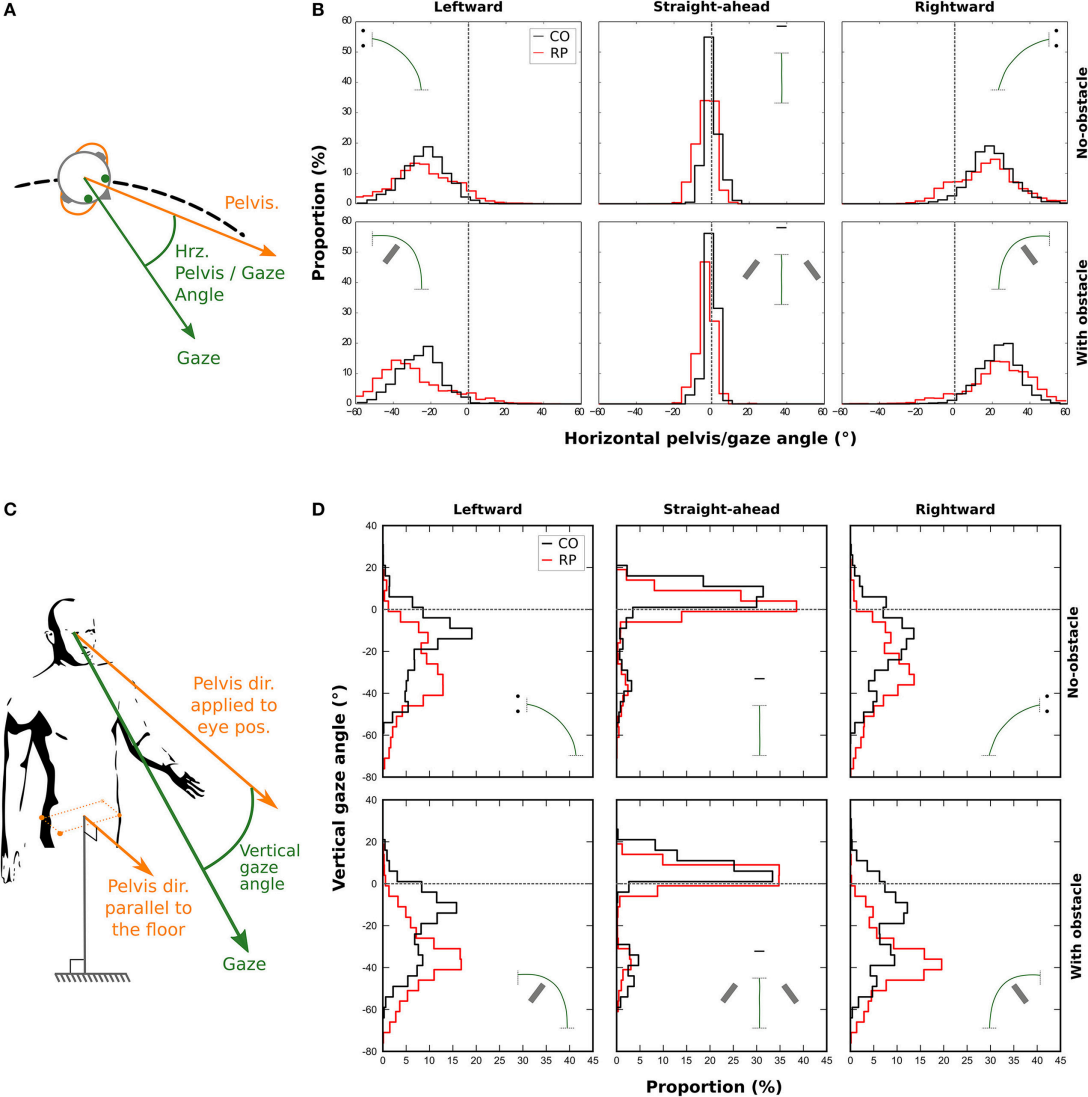
Distributions of the horizontal **(B)** and vertical **(D)** angle between gaze and pelvis directions during the trial for control (black line) and RP participants (red line). A positive horizontal angle is a clockwise rotation of the gaze away from the pelvis (see **A**). The vertical gaze angle is measured as an offset from the vertical component of the pelvis direction. **(C)** A negative vertical angle is a rotation of the gaze away below the horizon.

A significant change in the locomotion strategy, and especially a more extensive exploration of the environment in RP subjects, should translate into a differentiated movement of the gaze relative to the rest of the body. In addition to the variability of the eye position and of the head orientation, we computed the horizontal gaze distribution during walking. We considered the direction of pelvis trajectory as a reference (Authié et al., 2015), and then calculated, for each participant, the angle between pelvis and gaze directions (Figures 7A,B). Then, we computed the standard deviation of this angle for statistical analysis.

A three-way Anova showed that the presence of an obstacle did not influence the standard deviation of the distributions [*F*_(1, 16)_ = 0.29; *p* = 0.96]. However, we found that the distributions were wider in bending trajectories than in central ones [*F*_(2, 32)_ = 382.24, *p* = 4.6 × 10^−2*3*^; Straight-ahead: 1.86 ± 0.82°, Leftward: 11.19 ± 3.55°, Rightward: 11.25 ± 3.45°], and wider in RPs than in controls [*F*_(1, 16)_ = 26.12, *p* = 1.04 × 10^−4^; RP: 10.02 ± 2.04°, Controls: 6.18 ± 0.96°].

##### Vertical gaze angle

We also considered the vertical gaze distribution with respect to the pelvis direction, in a slightly different way. We defined a pelvis vector with a null vertical component (i.e., keeping only the actual horizontal pelvis direction, see Figure 7C). This vector was then applied to the eye position to compute the vertical gaze angle, which was computed for each participant (Figure 7D).

We computed the median of this vertical angle for statistical analysis. A three-way Anova showed no main effect—but a tendency—of the group, when considering all trajectory directions [*F*_(1, 16)_ = 2.47, *p* = 0.09]. However, participants from both groups looked more often downward, i.e., toward the floor, in the obstacle condition (−18.77 ± 11.60°) than in no-obstacle condition [−14.50 ± 8.94°; *F*_(1, 16)_ = 11.98, *p* = 0.003]. Moreover, the gaze was also more oriented downward in leftward (−25.29 ± 12.37°) and rightward (−25.80 ± 12.95°) trajectory directions, than when walking straight-ahead [1.18 ± 8.90°; *F*_(2, 32)_ = 106.82, *p* = 6.89 × 10^−15^]. Finally, an interaction between group and direction variables [*F*_(2, 32)_ = 4.69, *p* = 0.01] showed that RPs oriented more their gaze toward the floor than controls, only in leftward and rightward trajectory directions (RP: −30.99 ± 9.30°, Controls: −20.11 ± 13.67°). This result indicates that RPs looked in average at 2.7–4.2 m for controls—in front of them in bending trajectories.

Finally, we computed the standard deviation of this vertical angle. A three-way Anova showed that both obstacle [*F*_(1, 16)_ = 0.01; *p* = 0.97], and group variables [*F*_(1, 16)_ = 3.02; *p* = 0.10] did not influence the standard deviation of the distributions. However, the distributions were wider in bending trajectories than in central ones [*F*_(2, 32)_ = 5.16, *p* = 0.01].

**In summary**, when compared to controls, RPs exhibited a wider horizontal exploration of the environment, not only with eye and head movements, but also with gaze movements with respect to the pelvis. On the vertical axis, results indicate that when walking in a curved trajectory, RPs directed their gaze more downward than controls, in the direction of the floor.

#### Fixation location

Over all trials and conditions, we have detected 2,034 fixations during the preparation phase (803 for controls, 1,231 for RPs) and 9,210 fixations during the walking phase (3,729 for controls, 5,481 for RPs). To investigate whereto in the environment subjects directed their gaze, we defined seven categories of fixation location, i.e., the intersection between the gaze vector and the 3D environment (see Figures 2E,F and Materials and Methods Section). Categories were the following: (1) the front wall including the screen; (2) the floor; the door including four specific locations: (3) the proximal edge of the door; (4) the distal edge of the door; (5) the top edge of the door; (6) the door aperture; and (7) the obstacle. For each subject and experimental condition, the proportion of fixations in each category was counted. Leftward and rightward trajectory directions were merged for statistical analysis.

**Figure 8:**
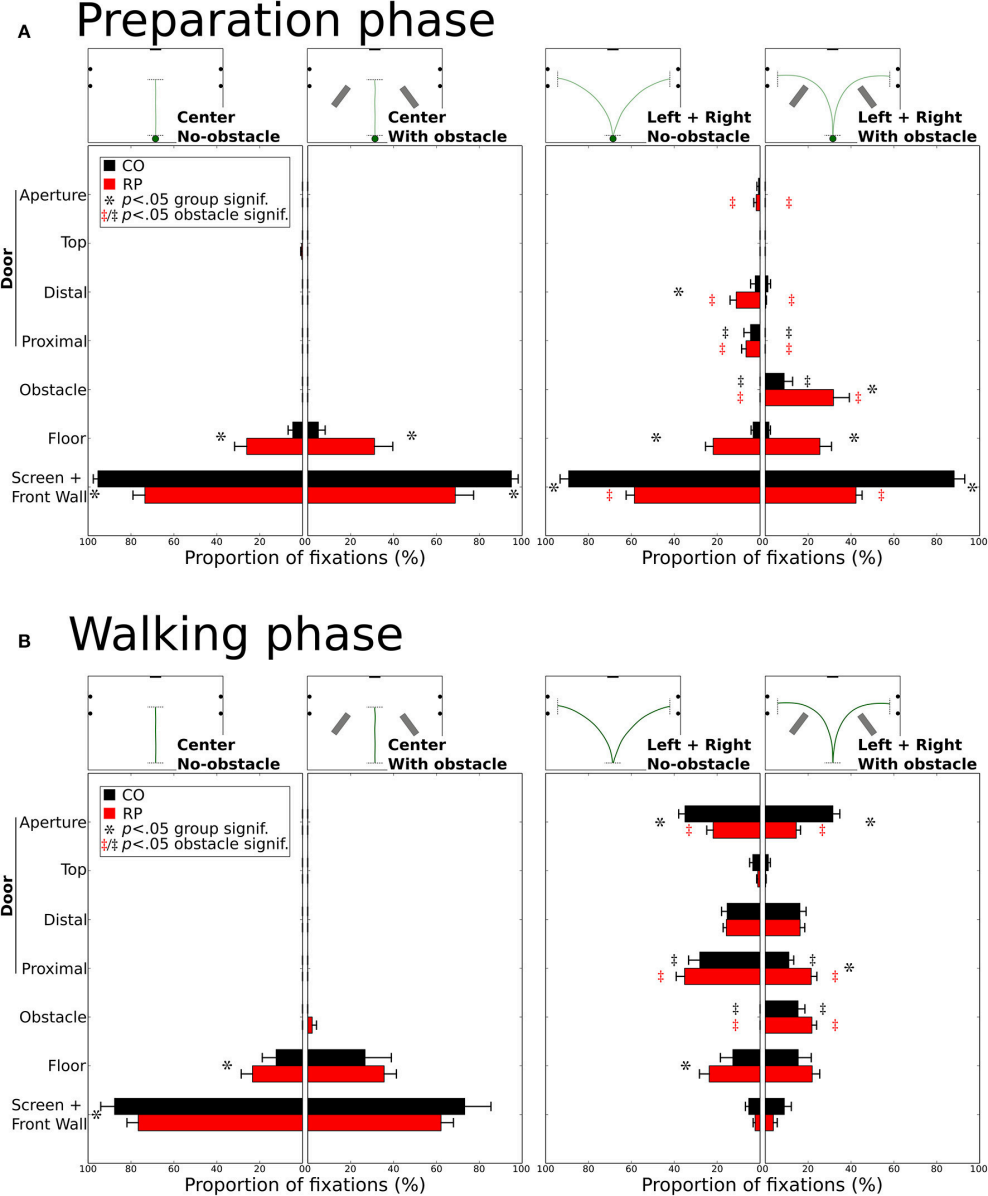
Distribution of the proportion of fixations for each gaze category during the preparation **(A)** and walking **(B)**. Mean proportions are represented for control and RP subjects, together with between-subjects standard error. Stars (^*^) indicate significant differences between groups within a gaze category, while double daggers indicate significant differences between obstacle conditions for the same group (RP ^
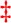
^ or CO ^‡^) and the same trajectory direction (center or leftward and rightward).

Figure 8 shows histograms of the proportion of fixations location per category during preparation (A) and walking phases (B). Mann–Whitney tests were only performed (i) between obstacle conditions for the same group, the same trajectory direction and the same fixation category; and (ii) between groups for the same obstacle condition, the same direction and the same fixation category. All significant differences are reported on Figure 8 and some of them will not be discussed in the text.

*During the preparation phase*, although subjects were already informed of the required direction to follow, most of their fixations were directed toward the front wall or to the screen (Figure 8A). RPs had a lower proportion of fixations on the front wall than controls in all conditions (all *p* < 0.01; CO: 92%; RP: 51%). Instead of mainly looking at the screen and the front wall, RPs also fixated the floor in the center trajectory direction, with and without obstacle (group difference *p* < 0.01), marginally the door in leftward/rightward conditions (group difference *p* < 0.05 for distal edge) and onto the obstacle when present in leftward/rightward conditions (group difference *p* < 0.05). In leftward/rightward conditions, when obstacles were present, RPs reduced their proportion of fixations on the front wall and the door (proximal and distal edges), and increased this proportion onto the floor and obstacles (*p* < 0.05, between conditions). Also, for controls, the presence of an obstacle in leftward/rightward conditions reduced the proportion of fixations on the distal part of the door, to look at the obstacles (all *p* < 0.01, between conditions).

*During the walking phase*, subjects walked toward a goal (central screen or door aperture). In the center direction without obstacles, RPs showed an increased proportion of fixations to the floor compared to controls (CO: 12%; RP: 23%; *p* < 0.01), and consequently a reduced proportion of fixations to the front wall (CO: 88%; RP: 77%; *p* < 0.001, Figure 8B). These differences were no longer significant with obstacles in the center trajectory direction, although some RP fixations (2%) were directed to obstacles. A larger diversity of fixation locations was observed in leftward/rightward conditions for both groups. With and without obstacles, the proportion of fixation in both groups was identical on the screen/front wall (4%) and the distal (16%) and top (3%) edges of the door. The proportion of fixations on the floor were also identical between obstacle conditions for both groups, but larger for RPs (*p* < 0.05; 24%) than controls (13%) without obstacle. As obviously expected, when obstacles were present, both RPs and controls allocated fixations onto the obstacles (CO: 25%; RP: 22%, *n.s*. between groups; *p* < 0.01 between obstacle for both groups). Fixation proportion was reduced for both groups on the proximal edges of the doors in the obstacle condition (*p* < 0.05), this reduction being lower for RPs (*p* < 0.05; no-obstacle/CO: 28%; obstacle/CO: 11%; no-obstacle/RP: 24%; obstacle/RP: 22%). Finally, the aperture of the door gathered a lower proportion of fixations in RPs than controls in both obstacle conditions (CO: 33%; RP: 18%; *p* < 0.01), this proportion being reduced only for RPs with obstacles (*p* < 0.05, 14%) as compared to no-obstacle condition (22%). Moreover, no significant correlation was found between the proportion of fixations on the obstacle or the floor, and the VF extent (with any VF measures, all *p* > 0.09).

**In summary**, before starting to walk, RPs demonstrated an exploratory behavior more pronounced than controls (including fixations on the floor, the door frame edges, and the obstacle). During locomotion, RPs directed their gaze to the floor and the door edges, with a peculiar pattern of alternated fixation on door edges, in contrast with controls who performed more fixations inside the door aperture. Finally, as expected, in obstacle conditions, participants form both groups allocated a number of fixations on obstacle edges, thus reducing the proportion of fixations to aperture and proximal edges for RPs, and to door aperture for controls.

#### Global strategy: evolution of fixation behavior during the trial

**Figure 9:**
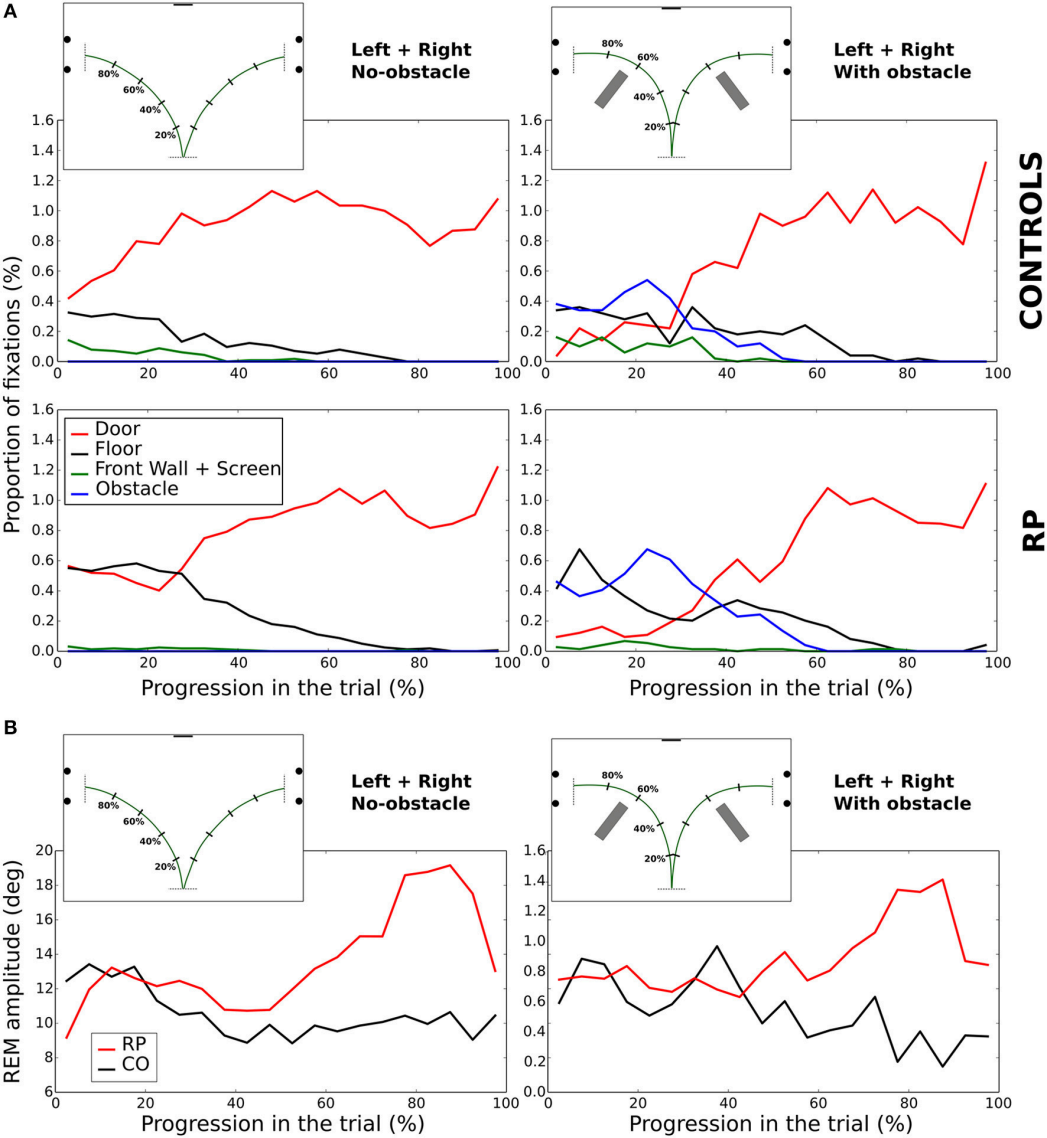
Evolution of fixations depending on the looked-at object **(A)**. Amplitude of REM **(B)** during the walking phase. In **(A,B)**, the trial length is divided into 5% bins.

In the previous section, we indicated *where at* subjects looked in the experimental room, but not *when* these fixations were performed during the trial. Figure 9A represents the average percentage of fixations directed toward four locations of interest (front wall with the screen, floor, doors, and obstacles) at each time interval and experimental condition (center trajectory excluded).

*In no-obstacle condition*, subjects from both groups fixated more often the doors at the end of the trajectory. In RPs, however, this behavior appeared only after completing ~27% of the trajectory, while it was observed all along the trial in controls. In the first quarter of the trial, RP fixations were equally distributed between floor and door, with floor fixations decreasing to become negligible around 80% of the trajectory. In controls, floor fixations were stable from the beginning to 80% of the trajectory when they become negligible, and a small proportion of their fixations were directed to the front wall and the screen before the 37% of the trajectory.

*With obstacles*, the proportion of fixations directed to the door was lower than in no-obstacle condition for both groups until ~32% of the trajectory, replaced by fixations toward the obstacles, with a clear peak for both groups at ~22%. RPs had also a fixation peak at ~7% onto the floor, a behavior not present in controls. After 52% of the trajectory, all subjects ceased to fixate obstacles, and the door was the most fixated category.

**In summary**, we observed some notable differences at the beginning of the trial, with a larger proportion of fixations on the floor for RPs, and on the task goal (the door) for controls. Otherwise, in both groups corresponding fixation patterns were performed roughly at the same normalized time.

#### Fixation strategies—illustrative cases

**Figure 10:**
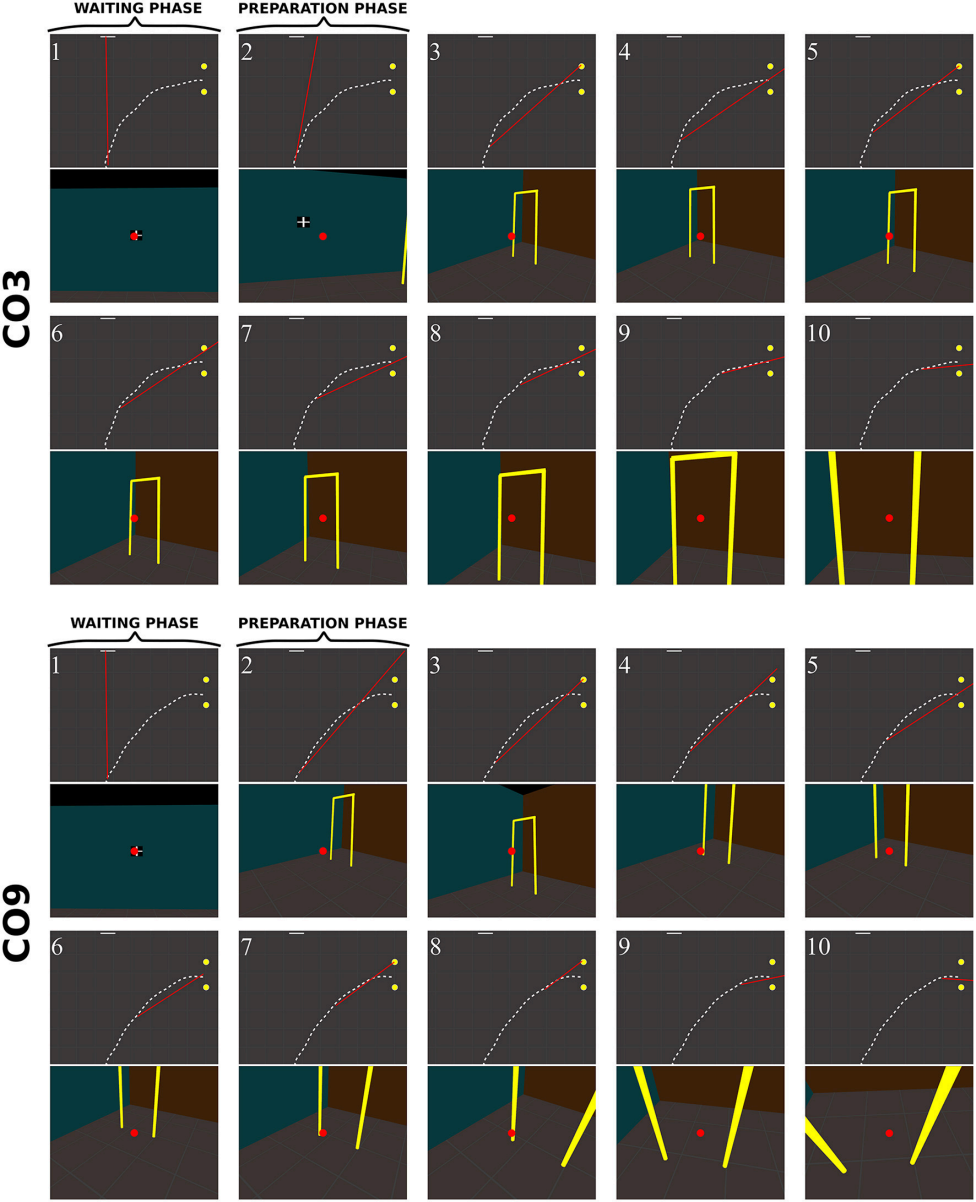
Sequence of fixations of two control subjects (top CO3, bottom CO9) in the right no-obstacle condition (see also Supplementary Video 1, https://www.youtube.com/watch?v=0-f0CuDcz1M&index=3&list=PLQ8v2CHny1mPTlAnEtUfzOkifV6gIwZA3). For each detected fixation, both top view (top) and perspective view (bottom) are represented. The **top views** display the whole trajectory of the head (dashed white line), the door (yellow disks), the frontal screen (white line) and the gaze direction (red segment). The **perspective views** show a reconstruction of the actual view from a camera (with a field-of-view of 60 × 72°) located at right eye position and oriented in the gaze direction, i.e., as it was successively visible in front of the participant. Gaze position is represented at the center of the image (red dot). The first fixation was performed during the waiting phase. The following two fixations were performed before the initiation of walking (12 cm from the starting point), during the preparation phase. The others were recorded while the subject walked toward the door. The last two fixations were performed in a path segment of 40 cm before the door position.

**Figure 11:**
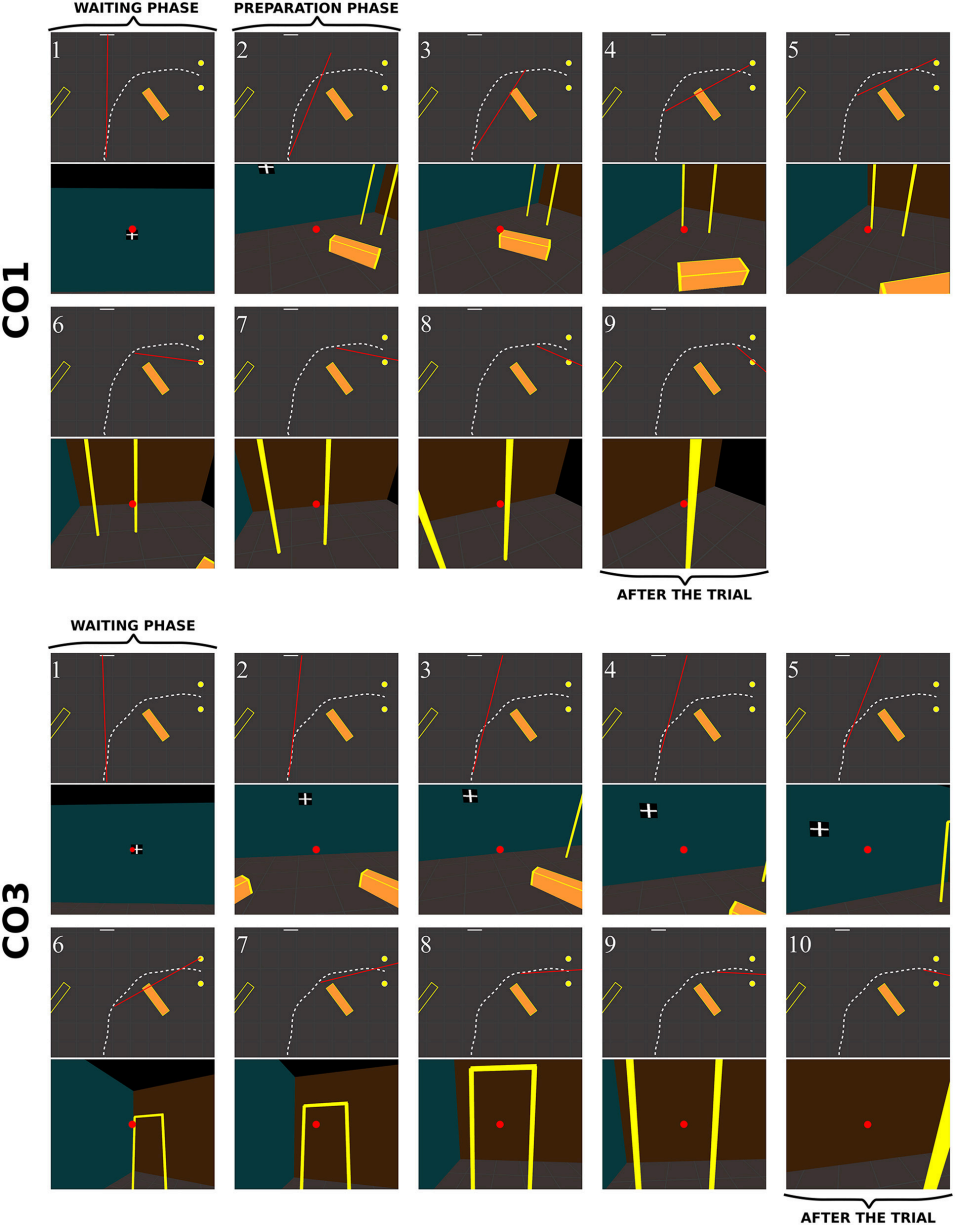
Sequence of fixations of two control subjects (**top** CO1, **bottom** CO3) in the right obstacle condition (see also Supplementary Video 2, https://www.youtube.com/watch?v=azN3B0hMj3s&index=4&list=PLQ8v2CHny1mPTlAnEtUfzOkifV6gIwZA3). The same conventions as in Figure 10 were adopted. Obstacles are represented as orange blocks with yellow edges.

**Figure 12:**
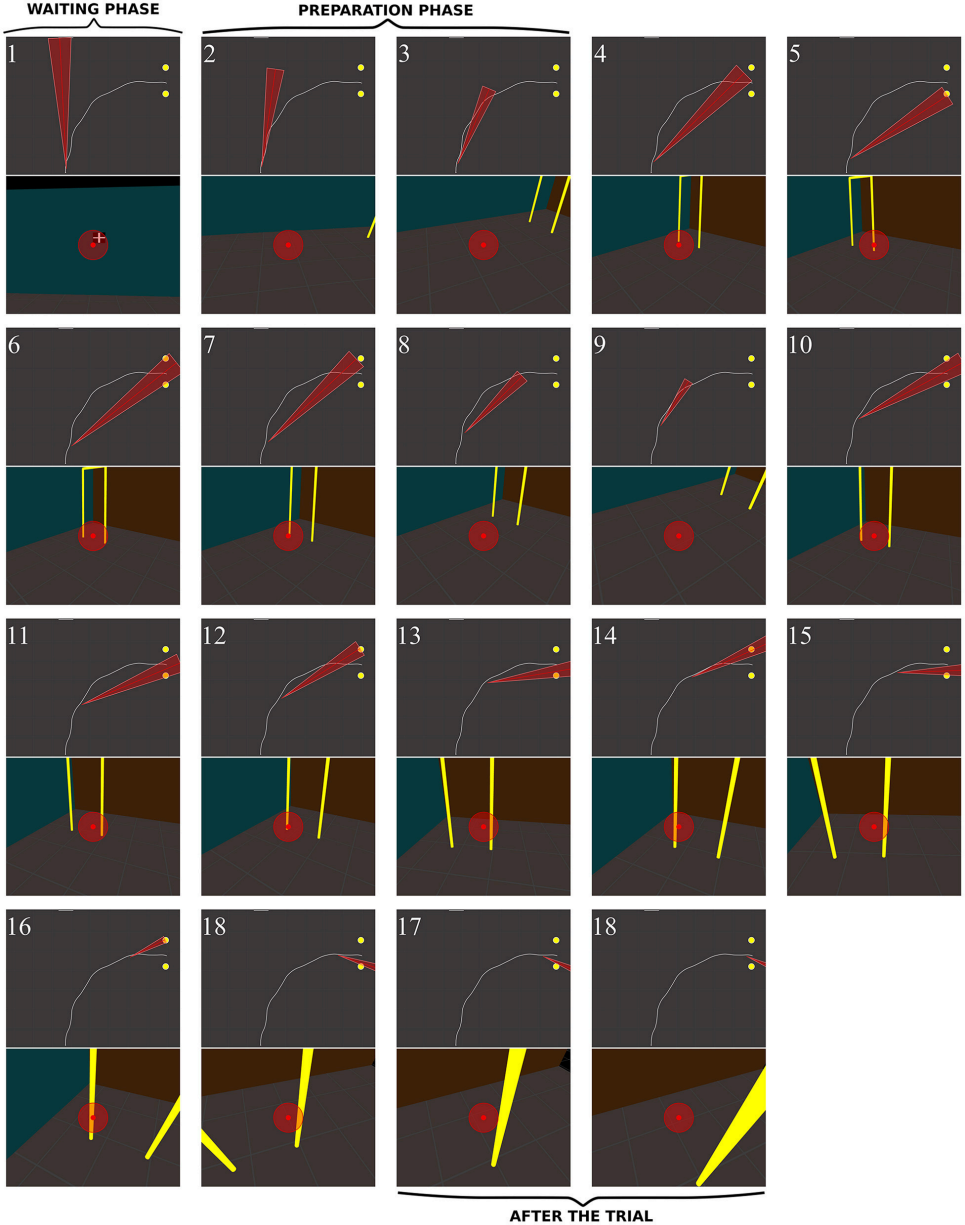
Sequence of fixations of one RP subject (RP6) in the right/no-obstacle condition (see also Supplementary Video 3, https://www.youtube.com/watch?v=JD3-vQ76YZs&index=2&list=PLQ8v2CHny1mPTlAnEtUfzOkifV6gIwZA3). The same conventions as in Figure 10 were adopted. On **perspective views**, gaze position is represented at the center of the image (red dot), as well as residual VF of the subject (red transparent disc, ~14°). The **top views** show the gaze direction (red segment) and the residual visual field (red triangle).

**Figure 13:**
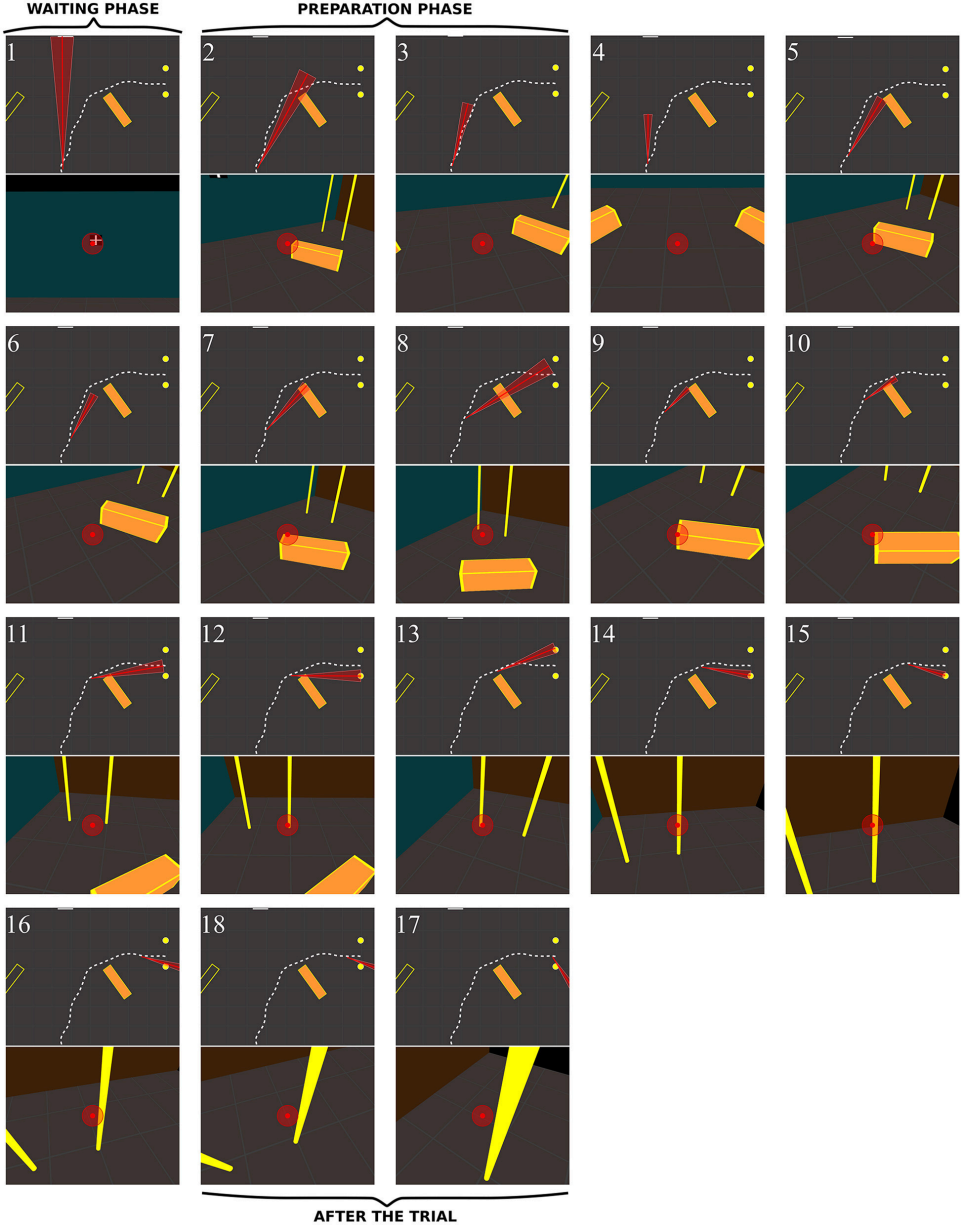
Sequence of fixations of one RP subject (RP3) in the right/obstacle condition (see also Supplementary Video 4, https://www.youtube.com/watch?v=Li03bCdRsZs&list=PLQ8v2CHny1mPTlAnEtUfzOkifV6gIwZA3).&index=1 The same conventions as in Figure 10 were adopted. The residual VF of the subject (~10°) is displayed as a red transparent disc on **perspective views**, and as a red triangle on **top views**.

A visual inspection showed that the sequences of fixation were qualitatively different between groups (Figures 10–**13**, see also Supplementary Videos 1–4: https://www.youtube.com/watch?v=Li03bCdRsZs&list=PLQ8v2CHny1mPTlAnEtUfzOkifV6gIwZA3), even though subjects from the same group occasionally exhibited different sequences of fixations during a trial.

##### Control subjects

Figure 10 shows successive fixations of two control subjects (CO3 and CO9) during a rightward trial without obstacle. Both subjects first fixated the distal edge of the door, and eventually the floor around the door (CO3: fix. 3–5; CO9: fix. 5–6). After completing 50% of the trajectory, subjects' behavior differed from each other. The first one mainly fixated the right wall through the door aperture (CO3, fix. 7–10). The second one fixated the floor inside the door aperture, and also the distal edge of the door. Both did not look on their future trajectory.

When an obstacle was present, controls also occasionally exhibited slightly different sequences of fixations (Figure 11). Some, as CO1, fixated the obstacle at the beginning of the trajectory (fix. 3), and others as CO3 did not perform any fixation on the obstacle. Before passing the obstacle, all subjects stared at the door, first on the distal edge (fix. 4 and 6 for CO1 and CO3, respectively), then either in the aperture (CO3) or the proximal edge of the door (CO1). As in no-obstacle condition, controls did not look at their future trajectory. Moreover, they also performed exploratory fixations, either on the floor or the front wall.

*In RPs*, the oculomotor behavior clearly differed from that of controls. Figure 12 shows the sequence of fixations of an RP patient (RP6, with a 14° residual VF) during a rightward no-obstacle trial. This subject first stared at the frontal screen, waiting for the instruction for the direction to follow (fix. 1). Following the instruction, and before starting locomotion, he directed two fixations onto the floor, including one on a location of the future trajectory, although he was informed that no obstacle was on his way. From the fourth to the eighteenth fixations, the subject repeatedly fixated the distal and proximal edges of the door; moreover, when the distance from the door allowed to include both door edges in the residual VF, he also looked at the ground of the door aperture. He then scanned locations on the future trajectory before the door, possibly to check that no obstacle were located on the floor. From the middle of his trajectory (fix. 11–18), he successively fixated door proximal and distal edges, with the ground always being in the VF, but never looked at the door aperture. At the end of the trajectory (last three fixations), he stared the proximal edge of the door, and then the ground around the future trajectory.

Figure 13 shows the behavior of a RP subject with a slightly smaller VF (RP3, 10°) with obstacle. Before starting to walk, he fixated the obstacle and explored his future trajectory. After starting to walk he fixated on or around the obstacle (fix. 5–7). The first fixation onto the door (fix. 8) was performed around 30% of the trajectory. Then, he fixated the obstacle (fix. 9–10) until the circumvention. By the end, his gaze strategy was similar to the one without obstacle (fix. 11–17), with alternated fixation on door edges, followed by fixations on the proximal edge, and finally on the ground around the future trajectory. Interestingly, between the eighth and the tenth fixations, while any door edges was in subject's VF, he was able to generate an accurate REM to the door (fix. 11), even though he had moved by one meter. Most fixations on the obstacle were located on its edges, including corners. We observed two main differences from controls: RPs performed more fixations for the same trajectory and did look at their future trajectory.

### Global summary

1. Peripheral VF loss lead to a more active visual exploration of the environment, with 28% more frequent REMs in RPs than in controls.
2. The REM rate was higher in RPs for eye movements between 15 and 29° in amplitude. Twenty-five percent of REMs were large enough to shift fixation beyond the limits of the patient's VF.
3. The range of eye movement scanning with respect to the head did not differ between groups, both vertically and horizontally.
4. RPs exhibited larger head movements than controls. In pitch, the head was more tilted forward in RPs than in controls, with larger movements when walking straight-ahead. In contrast, the average orientation of the head in yaw did not differ between groups.
5. All above findings resulted in a more extensive visual sampling in RPs, but on the same environmental features.
6. RPs exhibited a wider horizontal exploration of the environment with gaze movements, and directed their gaze lower (toward the floor) than controls.
7. RPs performed more frequent fixations on the floor (although knowing that no obstacles were located on their way), on door edges. When approaching the door, as their residual VF could not anymore include both door edges, RPs alternated fixations between proximal and distal edges.
8. The trajectory geometry did not differ between groups, with however a slightly smaller walking speed in RPs.

## Discussion

To assess the impact of peripheral VF loss on motor behavior during locomotion, we compared full-body, head, and gaze movements of normally-sighted and RP subjects with tunnel vision, during a goal-directed locomotor task, with or without obstacles.

Numerous changes in fixation pattern were observed, including enhanced visual sampling, proactive target selection, fixation patterns including downward fixations, and synergistic head movements allowing for a successful trajectory execution. Our observations suggest that reduced awareness of the visual environment, following peripheral VF loss generated adapted gaze strategies, in order to detect changes in spatial configuration, collect information for self-motion, update the postural reference frame, and update egocentric distances to environmental objects.

### Enhanced visual sampling in RP patients

RP subjects exhibited a 28% increase in REM rate, compared to normally-sighted individuals. This was however only observed in eye movements from 15 to 29° in amplitude. Moreover, 25% of REMs were large enough to generate a post-saccadic fixation located beyond the limits of patient's field of view before REM initiation. This occurred especially when the residual VF was small. In contrast, no difference was found between groups regarding the range of eye movement scanning with respect to the head (i.e., eye movement variability), both vertically and horizontally, and REM rate was not either affected by the presence of obstacles on the path. This absence of difference between groups was compensated with larger head movements in RPs, in a wider gaze exploration from the pelvis direction.

In a locomotion task performed by RPs, Luo et al. (2008) found an increase in REM rate similar to ours (2.67 vs. 2.92 REM/s). Other studies on visual search (Coeckelbergh et al., 2002b; Cornelissen et al., 2005; Smith et al., 2012b; Wiecek et al., 2012) and driving (Crabb et al., 2010), showed contradictory results. Diverging observations were presumably due to heterogeneity in considered diseases, and to differences in experimental settings, e.g., some studies involving actual VF defects while other investigations were based on simulated VF restrictions (Cornelissen et al., 2005).

A study by Vargas-Martin and Peli (2006), with RPs presenting a VF loss similar to that of our subjects, reported a reduced variability of eye position along the horizontal axis, but not along the vertical axis, when compared to normally-sighted subjects. The use however of long canes during their walking task, to monitor ground obstacles, could account for the difference in horizontal variability compared to our results.

The substantial differences in eye movements showed by RP subjects suggest an adaptive gaze strategy developed to optimize visual sampling in goal-oriented tasks with chronically restricted VF. The absence of peripheral VF constraints and reshapes the visual exploration.

### Proactive target selection

As previously noted, RP subjects performed a significant number of REMs, shifting fixation beyond VF borders. This behavior was amplified in the last phase of the trajectory, when subjects approached the door. In that phase of the trial, door edges appeared to the participant more and more (angularly) distant, requiring gradually larger saccades for being alternately fixated. This expressed an adaptive fixation pattern for on-line trajectory control.

As previously suggested (Land and Furneaux, 1997; Luo et al., 2008), REMs toward visual targets beyond the subject's field of view are proactive, as they are not elicited by salient visual stimuli. In normally-sighted subjects, target selection is performed through both salience driven (bottom-up; Peters et al., 2005; Foulsham and Underwood, 2008) and proactive goal driven (top-down; Land and Furneaux, 1997; Rothkopf et al., 2007) mechanisms. Indeed, the visual behavior in the control group is compatible with this hypothesis. We propose that proactive target selection is a central feature of visual behavior in patients with restricted VF.

### Adaptive fixation patterns in movement preparation and execution phases

In RPs, movement preparation and execution required a number of peculiar steps related to restricted VF.

During the *preparation phase*, RPs first explored the environment (i.e., the doors, the obstacles and the floor) while normally-sighted subjects immediately fixated straight-forward (i.e., the front wall and the screen). One could argue that the purpose of this behavior was obvious, namely addressing the RPs' need to explore the new environment and determine the relative position of visual clues and objects. Surprisingly however, with repeated trials in identical environments (e.g., in series of 38 trials without obstacle) affected individuals exhibited a similar exploratory behavior. In the preparation phase, these individuals could need to repeatedly update the spatial references (Turano et al., 2005) due to their peculiar visual awareness and attention. Indeed, while in normally-sighted, both covert and overt visual attention can be deployed for movement preparation, RP subjects essentially benefit from overt visual attention due to their VF restriction.

In contrast, in the *execution phase*, gaze fixations followed similar general patterns in all tested subjects. The latter spent most of the time looking at the final goal. This was previously described as a “look where you are going” strategy (Wann and Swapp, 2000), and reported in a variety of tasks, e.g. in driving situations (Land and Lee, 1994), but also with walking RP subjects (Turano et al., 2001). Moreover, the presence of obstacles lead both groups to often look at the obstacle at the beginning of the trajectory and reduce their proportion of fixations to the door during the trial. Finally, participants looked at the door during the final phase. This similarity between groups in fixation sequence needs to be investigated. At first glance, this behavior might appear in contradiction with the observations by Turano et al. (2001), showing a larger fixation area in RPs than in controls when walking in a hallway. One must keep in mind however that, in the present study, RPs performed more fixations than controls did, even though the proportion of fixations was comparable in both groups during walking, leaving aside the door approaching phase. Therefore, their visual exploration was larger, but fixations were directed to the same objects.

Moreover, during the walking phase with obstacle, the gaze behavior of both groups was divided in four successive sub-phases: (1) an obstacle approaching phase between the initiation and ~35% of the trajectory length, with fixations on the obstacle and the floor; (2) an obstacle circumvention phase until ~55% of the trajectory, with fixations on the floor and the door; (3) a door approaching phase until ~70% of the trajectory, with fixations mostly at the door; and (4) a crossing phase at the very end of the trajectory with fixations on the inside edge of the door. Gaze fixations were ~25% ahead of the body, or in other words one sub-phase ahead. This corresponded to 1 s anticipation in a 4 s trial (for instance between the obstacle fixation peak at 22%, and the obstacle circumvention at ~50%), matching closely delays reported during driving (Land and Lee, 1994). This behavior could correspond to a strategy maintaining the information gathered during the fixation in the short-term memory (Ballard et al., 1995). Moreover, interestingly, to update the goal location while moving, in the no-obstacle condition, RP subjects fixated the final goal sooner than controls.

The present results indicate how RP subjects tend to compensate for peripheral VF loss. They indirectly emphasize the importance of peripheral vision, not only to gain information on the egocentric position of environmental objects, but also to build-up and update the spatial relations between objects. As they move, affected individuals must perform systematic exploratory and confirmatory fixations (on the future trajectory, obstacles, and goals) to get updated locations of environmental features. Without such a robust strategy, RP subjects would probably accumulate localization errors. Indeed, Yamamoto and Philbeck (2013) showed that impeding eye movements by field-of-view restriction impaired the accuracy of spatial learning (see also Fortenbaugh et al., 2007, 2008; Legge et al., 2016). In addition, several studies indicated that peripheral VF loss does not interfere with the perception of egocentric distance and the direction of an object, when subjects can fixate the object (Ooi et al., 2001; Creem-Regehr et al., 2005; Gajewski et al., 2014). The act of performing a fixation of an object is sufficient to accurately perceive its distance (Gajewski et al., 2014), by combining the gaze declination from the horizon and an estimate of eye level (Ooi et al., 2001). Therefore, without access to environment visual perception, RPs apparently need to fixate more often surrounding objects.

The updating of relative object locations with gaze movements, that was noted when subjects approached the door, is an additional crucial aspect of strategical changes occurring in RP subjects. The visual angle subtended by proximal and distal edges then tends to be larger than that of the subject's VF (on average, when the subject distance from the door was below 0.8 m). In such a condition, subjects did not have access to either static (Sedgwick, 1980; Warren and Whang, 1987) or dynamic (Fath and Fajen, 2011) information about size and “passability” of the door. Therefore, the observed sequence of alternated fixations of door edges reflect the need of frequently determining door size and position, to steer locomotion in the aperture direction. A similar behavior was reported in normally-sighted subjects when using an unusual mode of locomotion—namely a wheelchair—to pass a frontal aperture (Higuchi et al., 2009), in order to attend both door edges and wheelchair. In the present study, when approaching the door, normally-sighted subjects preferred to look at the door aperture or at proximal edge of the door, so that both sides of the door were visible in their central and peripheral vision, as in Cinelli et al. (2009). Interestingly, at the very end of the trial, subjects of both groups essentially fixated the proximal edge. At that stage, the proximal edge represented the “visual pivot” (Ripoll et al., 1995) to circumvent and come back to the starting position for the next trial. This fixation pattern was compatible with previous research showing, in more natural situations, fixation toward the inside edge of the trajectory during driving (Land and Lee, 1994; Authié and Mestre, 2011) and walking (Bernardin et al., 2012; Authié et al., 2015).

### Increased downward fixations in RP group

Most interestingly, RP subjects exhibited an increased proportion of fixations directed to the floor, both in preparation and execution phases. This behavior also noted by Turano et al. (2001), and accompanied by synergistic head movements (see below), possibly reflected the additional cognitive load of ground exploration in these patients. It may be conditioned by the prolonged experience in obstacle detection and avoidance following the loss of peripheral vision (Turano et al., 2001). Although, obstacle avoidance was an obvious cause of visual exploration of the ground in walking RP subjects, we were surprised to note that this gaze behavior also occurred when RP participants knew that no obstacle was present in the setting.

We therefore propose that, in addition to the need of asserting free access to walk forward, affected subjects look at the floor to also acquire relevant visual information for postural and locomotor control, as ground has been demonstrated, in normally-sighted subjects, to be a stable external reference (i.e., an invariant source of information for Gibson, 1950). Thus, the ground surface is used as the dominant reference to determine the relative distances of objects in 3D scenes (Bian et al., 2006), and features a greater processing efficiency than other surfaces (e.g., walls) for the visual control of posture (Flückiger and Baumberger, 1988). In addition to the purely visual cues, ground surface provides crucial somatosensory information for the control of posture and for supplying a stable reference (Kluzik et al., 2007). We presume that in our experimental tasks, repeated ground fixations in RP subjects were also used to update a postural reference frame and to collect self-motion information for trajectory control.

This consideration prompted us to call back for an interview, one of the RP subjects who had taken part in our experiment, and who incidentally is a very keen observer. When asked why he so often looked at the ground, the patient first appeared unable to provide a clear reply. We eventually requested him to stand up, look at the ground and describe his observations. He said verbatim the following (translated form French): “*when I am looking at the ground I feel something intense is occurring inside my body, I feel deeply anchored into the ground, standing straight, linked to the ground, ready to go*.” Then when asked to describe what he noticed when looking at a 2-m distant wall in front of him, the patient said the following: “*Well*, …*that's not the same, it does not induce the same feeling as when looking downward.”* The patient further noted that “*anyway, when walking while gazing in front, [he] rapidly feels compelled to have to look at the ground.”* His observations thus proved to be illustrative of the potential mechanisms mentioned above.

Moreover, we propose a third hypothesis accounting for the more frequent downward fixations in RPs, which is compatible with the previous two explanations. Controlling human locomotion in a curved trajectory involves two concurrent visual feedbacks and motor controls: a visual anticipation of the future trajectory (the goal, in the direction of the trajectory and usually fed by central vision in normally-sighted participants) and a visual compensation of steering deviation from the desired path (usually fed by peripheral vision). This idea was initially proposed by Donges (1978) in the context of curve driving, and referred as the *two-level visual control model*. A large body of experimental results supports this hypothesis (e.g., Land and Horwood, 1995; Salvucci and Gray, 2004; Frissen and Mars, 2014). The visual compensation component is an online continuous process. Whereas, normally-sighted participants can rely on peripheral vision to perform online correction, RP participants cannot gather both visual pieces of information at a glance. They might therefore need to look at the ground to acquire the visual information necessary for online steering correction, and this could explain why they resorted to this strategy over trials.

### Synergistic head movements

As observed in other disorders affecting peripheral VF (Coeckelbergh et al., 2002a; Kasneci et al., 2014), our results showed that head movement patterns in RP differed from those of controls.

The head *yaw* (horizontal rotations) showed comparable mean orientation in both groups, but higher variability in RPs, for left and right trajectory directions, especially in affected individuals presenting a VF <15° in diameter. As a rule, head direction anticipates the torso direction, the latter anticipating trajectory direction, on the horizontal axis, as reported in several studies (Grasso et al., 1998; Bernardin et al., 2012; Belmonti et al., 2013). Also, head anticipation favors a global gaze orientation toward visual goals (Reed-Jones et al., 2009a,b; Mestre and Authié, 2012), and could contribute to trajectory planning (Berthoz, 1997; Grasso et al., 1998). In our RP subjects, changes in head direction were associated with larger REMs. Indeed, following the well-established principle of eye-head synergy (André-Deshays et al., 1988), a relevant contribution of head movements is more likely to occur with larger gaze shift (>20°; Freedman, 2008).

Note worthily, in *pitch* (vertical) rotation, RPs tilted more their head forward than controls in all experimental conditions, but with a higher variability; but in RPs, the variability was higher when performing the straight-ahead locomotion task, particularly for subjects with smaller VF. This behavior may be conditioned by the above-mentioned mechanisms of detection and avoidance of low-lying obstacles, of information collection for self-motion control, and for the updating of a postural reference frame. In straight-forward condition, increased head variability may reflect the two constraints of the task: to evaluate the distance from the screen, located at eye-height, and to check potential low-lying obstacles. A slight forward tilt of the head might facilitate the alternating gaze-check of the wall and the floor. As a consequence of this bi-modal gaze direction, head movements were more often performed around this global head orientation, subtended by natural eye-head coordination (Freedman, 2008). It is also conceivable that tilting the head forward reflects an adaptive strategy aiming at improving the sensitivity of the otoliths (Pozzo et al., 1990; Hirasaki et al., 1993), thus helping RP patients to integrate inertial information related to self-motion.

### Global trajectories

Average trajectory did not differ between groups, either with or without obstacles. Trajectory variability, between-subjects and between-trials, did not differ between groups, nor minimal distance from the obstacle.

It has been reported that the trajectory accuracy was altered when the VF was artificially restricted to 30° in diameter, and that the trajectory was broadened around obstacle with a VF reduced to 60 and 90° in diameter (Toet and Jansen, 2007; Jansen et al., 2011). Our results, however, did not show such broadening with our RP subjects. This discrepancy may result from the fact that former studies used simulated field constriction that did not fully reproduce the condition of subjects presenting actual chronic VF defects, in association with adaptive sensory-motor strategies found in subjects with chronic conditions.

The identical, stereotyped trajectories observed both in normally-sighted and RP subjects suggest a common planning mechanism responsible for path optimization (Todorov and Jordan, 1998; Bennequin et al., 2009; Pham and Bennequin, 2012). Our results are compatible with previous observations on stereotyped hand movement trajectories and locomotor paths (Hicheur et al., 2007; Pham and Hicheur, 2009; Jansen et al., 2011) across various lighting conditions (Pham et al., 2011). Moreover, the planning mechanism might be independent from visuomotor control, impaired by the peripheral VF loss.

As previously noted in RP (Geruschat et al., 1998), in bilateral glaucoma (Friedman et al., 2007) and in artificially restricted VF using goggles (Toet and Jansen, 2007; Jansen et al., 2011), our RP subjects walked more slowly than controls in all tested conditions (~9% reduction), and this may be a naturally increased caution (Jansen et al., 2011). The reduced walking speed could be caused by an over-estimation of walking speed in these patients. This hypothesis appears improbable as perception of visual speed was actually reported to be reduced with peripheral VF restriction (Pretto et al., 2009); moreover vection (i.e., subjective sensation of self-motion) is less elicited by central stimuli (Brandt et al., 1973; Berthoz et al., 1975; Held et al., 1975). Alternatively decreased walking speed in RPs might be related to a potential multi-sensorial re-weighing toward somesthetic cues during walking, as a reduced speed could increase the time for haptic exploration of the ground with the foot's plantar surface (Hallemans et al., 2010).

### Limitations

The generalization of the present study is limited by some characteristics of its experimental conditions: the setup environment is a bound and plain volume, with few colors and objects contrasting with the background. The trajectory is admittedly very small and therefore strongly constrained. Our results cannot be safely extrapolated to outdoor walking situations. The present experimental configuration is nevertheless representative of indoor situations, involving a daily life activity, achievable under sufficient lighting conditions.

## Conclusion

Overall, these results indicated that following VF loss and associated reduction in awareness of the visual environment, patients need for increased visual exploration. REM rate was increased, REMs were sometimes larger, even resulting in post-saccadic fixation beyond the limit of the VF. Occurrence of such REMs suggested that at least partly, target selection was proactive rather than simply reactive (e.g., to salient visual stimuli). To build-up, update the spatial referential (egocentric position and localization of environmental features), prepare the trajectory to follow, and control it online, RP subjects had developed a particular fixation pattern, including more frequent fixations at the floor, to monitor low-lying obstacles, and interestingly also to collect information for self-motion control and to update a postural reference frame. These adaptive gaze strategies induced a synergistic alteration in head movement. Such adaptive changes presumably allowed achieving trajectories that showed a stereotyped pattern, similar to that of normally-seeing individuals. We also noted that beside the overall characteristic trajectory observed in all affected subjects, the pattern of successive fixations was qualitatively different in each individual, and in each groups. This could reflect the occurrence of additional punctual strategies, some possibly being adapted to individual clinical features that could not be recognized in our investigation. Our findings, as well as ulterior, additional information on individual strategies using different, more elaborated tasks, are expected to provide invaluable information to optimize visual training programs.

## Author contributions

Conceived and designed the experiments: CA, AB, JS, AS. Performed the experiments: CA. Analyzed the data: CA. Contributed reagents/materials/analysis tools: CA. Wrote the paper: CA, AB, JS, AS.

### Conflict of interest statement

Commercial relationships: JS (Pixium Vision [C,I]; GenSight Biologics [C,I]; Sanofifovea [C]; and Genesignal [C], Vision Medicines [C], Chronocam [I]; Chronolife [I]). The other authors declare that the research was conducted in the absence of any commercial or financial relationships that could be construed as a potential conflict of interest.

## Footnotes

^1^In contrast to other conditions affecting peripheral VF, particularly glaucoma, RP as a rule generates a field loss that is well-delineated and more regular in pattern. As a result, RP is associated with VF changes that are relatively safe to evaluate, in their daily, practical implications.

^2^Snellen visual acuity is measured using a chart of eleven lines of block letters, decreasing in size. The perception of five out of six letters on a line is considered to be the Snellen fraction. Snellen standard vision (20/20) is the ability to recognize letters subtending 5 minutes of arc. LogMAR visual acuity corresponds to the logarithm of the minimum angle of resolution, 20/20 in Snellen corresponding to 0 LogMAR.

^3^To evaluate the importance of VF limitation, and its impact in actual testing conditions, we used both Goldmann perimetry, commonly used to describe VF size, and door frame presentation in the experimental lighting conditions. It appeared that these VF measurements are highly correlated (*R* = 0.86, *p* < 0.05) and therefore lead to similar results in most analyses. We believe, however, that the principle of our additional VF evaluation in real testing conditions was relevant, and we advise its use in future studies devoted to practical implications of VF defects, whenever applicable.
